# A Mixed Model for Assessing the Effect of Numerous Plant Species Interactions on Grassland Biodiversity and Ecosystem Function Relationships

**DOI:** 10.1007/s13253-022-00505-2

**Published:** 2022-09-01

**Authors:** Jack McDonnell, Thomas McKenna, Kathryn A. Yurkonis, Deirdre Hennessy, Rafael de Andrade Moral, Caroline Brophy

**Affiliations:** 1 Present Address: Dundalk Institute of Technology, Dundalk, Co. Louth Ireland; 2 Teagasc, Animal and Grassland Research and Innovation Centre, Moorepark, Fermoy, Co. Cork Ireland; 3 Maynooth University, Co. Kildare, Ireland; 4 Kansas Biological Survey, University of Kansas, Lawrence, KS USA; 5 Department of Biology, University of North Dakota, Grand Forks, ND USA; 6 Trinity College Dublin, Dublin 2, Ireland

**Keywords:** Diversity-Interactions modeling, Random effects, Tallgrass prairie, Variance–covariance structure, Weed invasion

## Abstract

In grassland ecosystems, it is well known that increasing plant species diversity can improve ecosystem functions (i.e., ecosystem responses), for example, by increasing productivity and reducing weed invasion. Diversity-Interactions models use species proportions and their interactions as predictors in a regression framework to assess biodiversity and ecosystem function relationships. However, it can be difficult to model numerous interactions if there are many species, and interactions may be temporally variable or dependent on spatial planting patterns. We developed a new Diversity-Interactions mixed model for jointly assessing many species interactions and within-plot species planting pattern over multiple years. We model pairwise interactions using a small number of fixed parameters that incorporate spatial effects and supplement this by including all pairwise interaction variables as random effects, each constrained to have the same variance within each year. The random effects are indexed by pairs of species within plots rather than a plot-level factor as is typical in mixed models, and capture remaining variation due to pairwise species interactions parsimoniously. We apply our novel methodology to three years of weed invasion data from a 16-species grassland experiment that manipulated plant species diversity and spatial planting pattern and test its statistical properties in a simulation study.Supplementary materials accompanying this paper appear online.

Supplementary materials for this article are available at 10.1007/s13253-022-00505-2.

## Introduction

The performance of many ecosystems can be improved by increasing species diversity in communities (Isbell et al. 2017). For example, in grassland ecosystems, responses such as community biomass production, weed suppression and soil carbon storage can be boosted by increased plant species diversity (e.g., Cardinale et al. 2011). Studies of the ‘biodiversity and ecosystem function’ (BEF) relationship explore biodiversity effects on ecosystem functions or responses. Many BEF modeling approaches (Hooper et al. 2005) focus primarily on the number of species (richness) as the driver of diversity effects; however, community plant diversity can also be characterized by the identity and proportions (evenness) of the resident species, and methods relying on richness alone cannot capture these other aspects of species diversity (Dooley et al. 2015). Diversity-Interactions (DI) models were introduced to facilitate the analysis of multi-species experiments using species proportions as predictors of ecosystem functions (Kirwan et al. 2007, 2009; Connolly et al. 2013; Dooley et al. 2015; Brophy et al. 2017). DI models enable users to estimate the contribution of individual species and their interactions with other species, in addition to richness, when assessing the effect of species diversity on ecosystem functions. However, when species richness is particularly high, it can be challenging to model numerous species interactions in this framework. Brophy et al. (2017) introduced methods to model a large number of species in a single year. Here, we develop this approach to apply to a repeated measures (multi-year) setting and incorporate methods to test how species interactions may vary across spatial planting pattern scenarios in experimental plots.

Our new methodology was motivated by the Species Pattern and Community Ecology (SPaCE) experiment, which is a plot-based grassland BEF experiment established with a pool of sixteen tallgrass prairie species. The experiment was designed to investigate the effects of biodiversity (richness and evenness) and spatial planting pattern on plant productivity and weed invasion (McKenna and Yurkonis 2016; McKenna et al. 2019). Monoculture (single species) and mixture plots of up to eight species were constructed from a pool of sixteen species drawn from four plant functional groups (warm-season grass, cool-season grass, forb, and legume). The sixteen species varied considerably within and across functional groups with respect to traits such as their growth habit, size, and phenology. The spatial pattern treatment involved planting species in a randomly dispersed fashion across regularly spaced locations in mixture plots or planting in aggregated groups of species; fine-scale species interactions may vary across these spatial planting patterns within plots. Weed biomass in each of three growing seasons (2012–2014) is the response we analyze here. The data presented three main statistical challenges each to be addressed in a repeated measures (multi-year) setting: (1) how to model the $$\left( {\begin{array}{*{20}c} 16\\ 2\\ \end{array} } \right) \, =$$ 120 pairwise species interactions in a biologically meaningful way; (2) how to assess the spatial planting pattern treatment that was applied to mixture, but not monoculture, plots; and (3) given the variety of species types in the study, how to adjust for heterogeneous variation across plots within each year.

In BEF studies, the extent to which plant communities resist invasion by undesired plant species is of particular interest. Undesired plant species (weeds) can reduce yields and forage quality in grasslands, and can prevent restored grasslands from reaching their diversity goals (e.g., Corbin and D’Antonio 2012). Weed invasion is a source of inefficiency and must be reduced to make grassland production systems more economically and environmentally sustainable. Herbicide use to control weeds is expensive (DiTomaso 2000) and can negatively affect the environment and human health (Mahanty et al. 2017). Combining multiple species in grassland ecosystems offers a solution that is less economically and environmentally expensive, since it can reduce weed invasion by making better use of plant-available resources (Maron and Marler 2008; Connolly et al. 2018).

By definition, plants interact and acquire resources over finite distances, and traditional ways of capturing species interactions within BEF models may not be sufficient for capturing changes in species interactions that come with changes in species richness, evenness, and planting patterns in communities (Yurkonis 2013). To test this effect, researchers alter within plot species planting patterns and have found that fine-scale species patterns affect community scale productivity and weed invasion responses (Yurkonis et al. 2012; Zhang et al. 2014; McKenna and Yurkonis 2016; Seahra et al. 2016; McKenna et al. 2019). However, these spatial planting patterns and their subsequent effects on species interactions have yet to be incorporated into BEF models. To address the role that fine-scale species interactions play in affecting community-scale responses and to resolve the scales over which plant species affect their communities, we need BEF models that incorporate tests for species interactions that are informed by the spatial relationships among plant species in manipulated communities.

In this paper, we develop a novel Diversity-Interactions (DI) model that addresses the statistical challenges presented in the SPaCE experiment dataset. We build on previous versions of DI models by developing a method to integrate a treatment (spatial pattern) that only applies to mixture (and not monoculture) communities, allowing for heterogeneous variance across plots in the variance–covariance structure of the model, and introducing novel, multi-year random pairwise interaction effects to handle large numbers of possible pairwise species’ interactions. The random effects included are highly unusual in that they are indexed by pairs of species and year, rather than by a plot-level factor, which is typical in mixed models. They supplement a low degree of freedom fixed-effect description of the pairwise species interactions to acknowledge that there may be additional variation due to pairwise species interactions that cannot be captured by biologically meaningful fixed effects. For each year, a new random effect is introduced for each pair of species (120 pairs in each year, totaling 360 random effects), and in each year they are assumed to have the same variances. Thus, only one variance parameter per year is required for the inclusion of the random effects. To support this effort, we present a simulation study to explore the statistical properties and test the limitations of the random effects approach to modeling pairwise interactions. Our approach increases the capacity for researchers to assess more nuanced BEF relationships and further investigate the spatial and temporal scaling of BEF responses.

## Data and Methods

### Review of Diversity-Interactions Models

Biodiversity and ecosystem function experiments aim to investigate how varying species diversity affects community scale responses (the ecosystem function). There is a long-standing history in BEF research to focus on species richness (number of species) as the driver of ecosystem functions (Byrnes et al. 2014), but evenness (how equally distributed the species’ relative abundances are) may also be strongly influential (Kirwan et al. 2007; Wilsey and Stirling 2007). While it is common to find a positive and saturating relationship between ecosystem function and richness (e.g., Scherber et al. 2010), variation around the (ecosystem function—richness) line may be attributed to the identities and the relative abundances of the species and interactions among them. Diversity-Interactions (DI) models (Kirwan et al. 2007, 2009) were developed to model data from BEF experiments in which species diversity is manipulated. However, DI models can account for variation attributed to species’ identities, species’ relative abundances, interactions among species and evenness, *in addition to* species richness. DI models use species proportions and their interactions to model ecosystem functions and were developed from earlier work by Scheffe (1963) and Cornell (2002). DI models are a form of response surface models (Box and Draper 2007), where the predictors are the species proportions that sum to one for each experimental unit and collectively form a simplex space. If the experimental design provides sufficient coverage around the simplex space, DI models can be used to predict for any community combination of relative abundances within the species pool, not just the exact communities that were included in the design, providing a major advantage over some other modeling approaches used in BEF research. Since their original development (Kirwan et al. 2007, 2009), the family of DI models has grown to facilitate many of the complexities that arise with data from biodiversity experiments, such as multivariate responses (Dooley et al. 2015), nonlinearity in the form of species interactions (Connolly et al. 2013), the modeling of a large numbers of species interactions in a single year (Brophy et al. 2017) and the modeling of interactions among phylogenetically diverse communities (Connolly et al. 2011), where phylogenetic diversity is a measure of species’ ancestral relationships. The approach has been applied to data from a wide range of ecosystem types in addition to grasslands, including bacterial communities (Connolly et al. 2013) and dung fauna diversity studies (O’Hea et al. 2010). The DImodels R package fits DI models to BEF data collected from a single site in a single year (Moral et al. 2021).

DI models generally take the form (Kirwan et al. 2009):

1 $$\begin{aligned} y=\hbox {Identities}+\hbox {Interactions}+\hbox {Structures}+\varepsilon \end{aligned}$$

The community-level response *y* is an ecosystem function, such as biomass in a grassland community. The species ‘identities’ enter the model as the species proportions ($$P_{i})$$, the ‘interactions’ enter as products of species proportions, while ‘structures’ are other experimental design structures such as block or treatments. The sum of the species proportions is one for each experimental unit. For example, with a pool of *s* species, a DI model may take the form:

2 $$\begin{aligned} y_{ml}=\sum _{i=1}^s {\beta _{i}P_{im}} +\sum _{1\le i<j\le s} {\delta _{ij}P_{im}P_{jm}} +\alpha _{l}+\varepsilon _{ml} \end{aligned}$$

where $y_{ml}$ is the ecosystem function for experimental unit *m* in block *l* and $\varepsilon _{ml}\sim N(0,\sigma ^{2})$. In monoculture (species *i* is the only species present), $P_{i}=1$ and all other proportions are equal to zero and the expected response for the *l*th block is ${E\left[ Y \right] =\beta }_{i}+\alpha _{l}$. The interaction potential between species *i* and *j* is $\delta _{ij}$. The $E\left[ Y \right]$ in a mixture is a weighted sum of the identity effects of each of the species present in the mixture, plus the combined pairwise interactions. Model (2) is the ‘full’ pairwise interactions model and it accounts for the effects of species identities and individual pairwise interactions between species on the response of interest. If there is a small number of species in the species pool, model (2) is a reasonable model to fit. However, when the species pool is large, model (2) is generally not of interest: the number of pairwise interactions is either too large to be biologically informative, or it may not be possible to estimate all pairwise interactions due to the experimental design (Brophy et al. 2017). For example, in a four-species system there are $$\left( {\begin{array}{*{20}c} 4\\ 2\\ \end{array} } \right) \, =$$ 6 pairwise interactions, while in a sixteen-species system there are $$\left( {\begin{array}{*{20}c} 16\\ 2\\ \end{array} } \right) \, =$$ 120 pairwise interactions. However, there are many biologically informative ways to simplify model (2), for example, there may be no interaction effects (identity model), or it may be assumed that all pairwise interaction terms are equal (average pairwise model), or constraints among interactions may be introduced according to biological functional groupings (functional group model) as detailed in Table 1 [and in Kirwan et al. (2009)]. Higher-order interactions may also be needed (Kirwan et al. 2009).

**Table 1:** Various forms of the pairwise species interactions component of Diversity-Interactions models (Kirwan et al. 2009)

Model name	Description of interactions (for *S* species categorized into *T* functional groups)	Interactions
Identity	No interactions	
Average pairwise	All pairwise interactions are equal; there is one interaction ($\delta )$ term	${\tiny \delta \sum \limits _{{1\le i<j\le s}}^s P_{i} P_{j}}$
Functional group (FG)	Assume *T* functional groups (FG$$_{1}$$ - FG$$_{T})$$, each with $$n_{t}$$ species, where $$t=1,\,\ldots ,\, T.$$ The parameter $$\omega _{qq}$$ is the interaction between two species from functional group *q* and $$\omega _{qr}$$ is the interaction between two species from different functional groups, i.e., where $$q \ne r$$. There are T $$+ \left( {\begin{array}{c} T\\ 2\\ \end{array} } \right) $$ interaction $$(\omega )$$ terms	$$\sum \limits _{q=1}^T {\omega _{qq}\sum \limits _{\begin{array}{l} { i,j\in {FG}_{k} }\\ { i<j }\\ \end{array}} {P_{i}P_{j}} } +\sum \limits _{\tiny \begin{array}{l} 1\le q<r\le T\\ \end{array}}^T {\omega _{qr}\sum \limits _{i\in {FG}_{q}} \sum \limits _{j\in {FG}_{r}} {P_{i}P_{j}} } $$
Additive species	The contribution of species *i* to a pairwise interaction ${(\lambda }_{i})$ is an additive constant, regardless of the species it is interacting with. The strength of the pairwise interaction between two species is the sum of the individual contributions of each species. There are *S* interaction ($\lambda )$ terms	$\sum \limits _{{1\le i<j\le s}}^s {{(\lambda }_{i}+\lambda _{j})P_{i}P_{j}}$
Full pairwise	All pairs of species interact uniquely; there are $$\left( {\begin{array}{*{20}c} S\\ 2\\ \end{array} } \right) $$ interaction ($\delta )$ terms	$\sum \limits _{{{1\le i<j\le s}}}^s {\delta _{ij}P_{i}P_{j}}$

### Experiment

The data used in this study were collected from the Species Pattern and Community Ecology (SPaCE) experiment at the University of North Dakota’s Mekinock Field Station (Mekinock, ND, USA) from 2012 to 2014, inclusive (McKenna and Yurkonis 2016). There were 170 plots ($1 \times 1 \hbox { m}$) arranged in a randomized block design consisting of five blocks. Each plot was divided evenly into an $8\times 8$ grid where each of the 64 cells was planted with a 16-week-old greenhouse grown plant at the beginning of the growing season in 2012. Sixteen tallgrass prairie species were planted in the plots with either 1 (monoculture), or 2, 4 or 8 (mixtures) species in each, with varying relative abundances at each richness level in mixtures. A spatial treatment with two levels was manipulated across mixture plots (those with more than one species). Species were either assigned randomly to single planting positions in the $8 \times 8$ grid (dispersed) or assigned randomly to four adjacent planting positions, forming $2 \times 2$ conspecific patches in the $8 \times 8$ grid (aggregated) (supplementary materials S.1). Non-focal species (weeds) were removed monthly by hand and collected, dried and weighed. Total weed biomass removed (g) in the growing season of each year is analyzed in this paper. The proportion of biomass of the planted species in each plot was recorded at the end of every growing season, providing annual ‘realized’ proportions (McKenna et al. 2019).

Plants with similar traits can be classified by their functional group (FG). In this experiment, there were four species from each of four plant functional groups: warm-season grasses, species 1 to 4: *Andropogon gerardii *(big bluestem), *Schizachyrium scoparium *(little bluestem), *Sorghastrum nutans* (Indian grass), and *Panicum virgatum *(switchgrass); cool-season grasses, species 5 to 8: *Elymus canadensis* (Canada wildrye), *Elymus trachycaulus *(slender wheatgrass), *Pascopyrum smithii* (western wheatgrass), and *Nassella viridula* (green needle grass); forbs, species 9 to 12: *Monarda fistulosa* (wild bergamot), *Solidago rigida* (stiff goldenrod), *Helianthus maximiliani* (Maximilian sunflower), and *Ratibida columnifera* (yellow coneflower) and legumes, species 13 to 16: *Desmodium canadense* (showy tick trefoil),* Astragalus canadensis* (Canada milkvetch), *Dalea purpurea* (purple prairie clover), and *Glycyrrhiza lepidota* (American licorice). Two-species plots contained a grass and either a forb or a legume, four-species plots contained one species from each FG, and eight-species plots contained two species from each FG. The species were randomly selected from the FGs for each plot according to these constraints.

### Description of New Methods

In the SPaCE data, there are 16 species and $$\left( {\begin{array}{*{20}c} 16\\ 2\\ \end{array} } \right) \, =$$ 120 pairwise interactions. Estimating all 120 pairwise interactions is not of interest here since their large number would be devoid of biological meaning, and not possible given the design of the experiment (there is partial confounding among pairwise interactions). Over the three years, we aim to describe the interaction effects using fixed effects as parsimoniously as possible, and to test the inclusion of random pairwise interactions to identify if any variation due to pairwise interactions remains unexplained in each year. We describe the model in this section, assuming that all pairwise interactions are equal (Table 1, average pairwise model), while in Sects. 2.4 and 3.2 we describe the full model fitting process and final choice of model, respectively. The model for the SPaCE data (3 years of repeated measurements on 170 plots) can be written as:

3 $$\begin{aligned} {\varvec{y}}={\varvec{X}}{\varvec{\beta }} +{\varvec{Zu}}+{\varvec{\varepsilon }} \end{aligned}$$

The predictors in the ${\varvec{X}}$ matrix include block effects, species proportions ($P_{i},\, i=1,\ldots ,16)$, and the single interaction variable computed as the sum of all pairwise interactions crossed with the spatial planting pattern treatment. The error term ${\varvec{\varepsilon }} \, \sim \, N\left( \mathbf {0},{\varvec{R}} \right)$, where ${\varvec{R}}$ is a ($510\times 510$) block diagonal matrix with $3 \times 3$ blocks for the repeated measurements on each plot. The diagonal blocks in ${\varvec{R}}$ can be the same across all plots or can differ based on plot characteristics, such as whether the plot is a mixture or monoculture.

For species $i = 1,\ldots ,15,$ $j = 2,\ldots ,16$, $i<j$, plot $m = 1,\ldots ,170$, and year $k = 1,2,3$:

$$\begin{aligned}&\displaystyle \left[ {510\, \times \, 360;\, {PP}_{ijmk}=\, P}_{imk}P_{jmk} \right] \mathbf {\, }{\, \times \, [360\, \times 1;\, d}_{ijk}]\\&\displaystyle {\varvec{Zu}}=\left( {\begin{array}{*{20}c} {\begin{array}{*{20}c} {PP}_{1,2,1,1} &{} 0 &{} 0\\ 0 &{} {PP}_{1,2,1,2} &{} 0\\ 0 &{} 0 &{} {PP}_{1,2,1,3}\\ \end{array} } &{} \cdots &{} {\begin{array}{*{20}c} {PP}_{15,16,1,1} &{} 0 &{} 0\\ 0 &{} {PP}_{15,16,1,2} &{} 0\\ 0 &{} 0 &{} {PP}_{15,16,1,3}\\ \end{array} }\\ \vdots &{} \ddots &{} \vdots \\ {\begin{array}{*{20}c} {PP}_{1,2,170,1} &{} 0 &{} 0\\ 0 &{} {PP}_{1,2,170,2} &{} 0\\ 0 &{} 0 &{} {PP}_{1,2,170,3}\\ \end{array} } &{} \cdots &{} {\begin{array}{*{20}c} {PP}_{15,16,170,1} &{} 0 &{} 0\\ 0 &{} {PP}_{15,16,170,2} &{} 0\\ 0 &{} 0 &{} {PP}_{15,16,170,3}\\ \end{array} }\\ \end{array} } \right) \left( {\begin{array}{*{20}c} {\begin{array}{*{20}c} d_{1,2,1}\\ d_{1,2,2}\\ d_{1,2,3}\\ \end{array} }\\ \vdots \\ {\begin{array}{*{20}c} d_{15,16,1}\\ d_{15,16,2}\\ d_{15,16,3}\\ \end{array} }\\ \end{array} } \right) \, \end{aligned}$$

i.e., $${\varvec{Z}}$$ is a 510 by 360 matrix which contains the 120 pairwise interactions ($$P_{i}P_{j})$$ separated into yearly columns (with zeros outside of the current year). It is assumed $${\varvec{u\, }}\sim \, N\left( \mathbf {0},{\varvec{G}} \right) $$, where $${\varvec{G}}=Bdiag({\varvec{M}}_{\mathbf {1,2}},\, \ldots ,{\varvec{M}}_{\mathbf {15,16}})$$ is a 360 x 360 block-diagonal (the operator *Bdiag* represents a block-diagonal matrix), with zeros outside the $${\varvec{M}}_{{\varvec{i,j}}}$$ blocks, and $${\varvec{M}}_{{\varvec{i,j}}}{=}\left( {\begin{array}{*{20}c} \sigma _{1}^{2} &{} 0 &{} 0\\ 0 &{} \sigma _{2}^{2} &{} 0\\ 0 &{} 0 &{} \sigma _{3}^{2}\\ \end{array} } \right) $$ is a $$3 \times 3$$ block solely indexed by year (for each *i*, *j*), i.e. there are 120 random effects included in each year, but they are constrained to have equal variance, thus there is only one variance parameter per year; it would also be reasonable to include nonzero covariances in the off-diagonals of the $${\varvec{M}}_{{\varvec{i,j}}}$$ matrices. The purpose of the variance parameter in each year is to test if there is variability due to pairwise interactions, additional to the fixed interaction effect terms: for example, for the average pairwise interaction model ($$\delta _{ijk}=\delta _{k})$$, $$\sigma _{k}^{2}$$ measures variation in the true $$\delta _{ijk}$$ around $$\delta _{k}$$, if it exists. Indexing the random effects $${\, d}_{ijk}$$ by species pair *i*, *j* (and year *k*) is unusual, since random effects are typically indexed by a plot-level factor such as a block. It is more common that the experimental or sampling design generates observations grouped according to one or more factors (which may be crossed or nested), yielding a hierarchical modeling structure in which observations within the same group are correlated. Hence, in our modeling framework the $${\varvec{Z}}$$ matrix differs to that expected by standard mixed model fitting software, such as nlme (Pinheiro et al. 2018) and lme4 (Bates et al. 2015) in R (R Core Team 2020) for example.

In the SPaCE experimental design, the spatial pattern treatment (aggregated or dispersed) is applied only to mixture plots (it is not possible to apply it to monocultures). The spatial pattern treatment can interact with the species interaction effect terms, allowing the fixed average pairwise interaction of all pairs of species to differ for aggregated and dispersed plots, i.e., pairs of species may interact differently depending on the spatial planting pattern. This could be due to higher intraspecific interactions (between individuals of the same species) and lower interspecific interactions (between individuals of different species) in aggregated plots than dispersed plots (Stoll and Prati 2001). Incorporating the spatial pattern treatment, model (3) can be written as

4 $$\begin{aligned} y_{klmn}=\mathrm \! \sum _{i=1}^s {\beta _{ik}P_{ikm}} +\delta _{kn}\! \sum _{1\le i<j\le s} {P_{ikm}P_{jkm}} +\! \sum _{1\le i<j\le s}{\!}{d_{ijk}P_{ikm}P_{jkm}} +\alpha _{kl}+\varepsilon _{klmn} \end{aligned}$$

where $$\alpha _{kl}$$ is block effect *l* in year *k*, $$P_{ikm}$$ is the proportion of species *i* in plot *m* relevant to the year *k* (i.e., planted proportion when $$k =$$ 1 and the proportion in the preceding year when $$k>$$ 1), and $${\varvec{\varepsilon }} \,\sim \, N\left( \mathbf {0},{\varvec{R}} \right) $$ as in Eq. (3). When $$P_{i}=1$$, $$\beta _{ik}$$ is the expected weed biomass for a monoculture of species *i* in year *k*, and when $$P_{i}<1$$, then $$\beta _{ik}P_{ik}$$ is the species identity effect contribution to a mixture. The parameter $$\delta _{kn}$$ is the fixed average pairwise interaction between species *i* and *j* in year *k* for spatial pattern *n*, where *n* can be either 1 $$=$$ aggregated or 2 $$=$$ dispersed. The fixed interaction term assumes that all 120 $$\delta _{ijkn}$$ pairwise interactions are equal to $$\delta _{kn}$$. This may not be sufficient, and if random pairwise interaction ($$d_{ijk})$$ terms are needed in year *k*, this acknowledges there is additional variation around $$\delta _{kn}$$ across all pairwise interactions. The random effect variance terms will be incorporated into fixed effects standard errors, improving inference.

Fitting 360 random effects with a constrained variance in each year is not a trivial coding challenge. We used SAS software version 9.4 (SAS Institute, Cary, North Carolina, USA) to fit our models, utilizing the LIN covariance structure in proc mixed, which allows user-defined variance–covariance matrix structures for random effects. Proc mixed uses a Newton–Raphson algorithm to maximize the likelihood function. To resolve convergence issues, Fisher scoring was used and starting values for variance parameters were specified. It is not possible to fit the models in the standard mixed model packages in R [e.g. LME4 (Bates et al. 2015)] since random effects in these packages are always indexed by a plot level factor such as block. However, we have fitted the multi-year model as defined in Eq. (4) by writing the log-likelihood manually and maximizing it using optimx in R (Nash and Varadhan 2011; R Core Team 2020). Supplementary materials S.2 provides a tutorial style guide to fitting the model in Eq. (4) to simulated data in SAS and R.

### Model Fitting Process for the SPaCE Experiment Data

Diversity-Interactions models were fitted to the three years of SPaCE data with total yearly weed biomass as the response. Proportions planted were used as predictors in year 1 and ‘realized’ proportions in the preceding year were used in years 2 and 3 (McKenna et al. 2019). The model selection process steps were:

1. Fixed effects selection: the identity, average pairwise, functional group and additive species models (each described in Table 1) were fitted by maximum likelihood (ML) assuming an unstructured variance–covariance structure and compared using likelihood ratio tests (LRTs) to select the best model.
2. Repeated measures variance–covariance structure: the fixed effects model identified in step 1 was used as a ‘baseline’ model and different variance–covariance structures were fitted by restricted maximum likelihood (REML) to account for repeated measures over years: compound symmetry, first-order auto-regressive and unstructured were compared using LRTs (Littell et al. 2006). To test for heterogeneity across plots, the block diagonal matrices in the ${\varvec{R}}$ matrix were fitted as: (a) constant across all 170 plots, (b) different for monocultures and mixtures, and (c) different for all FGs in monocultures, and mixtures; these models were compared using LRTs.
3. Fixed effects and variance–covariance structure: if the variance–covariance structure was changed in step 2, step 1 was repeated, using the new error structure. Also, the species interaction terms were tested for interaction with spatial pattern and given their potential influence, legume percentage. Models were fitted by ML and compared using LRTs.
4. Random pairwise interactions: the model chosen in step 3 was fitted using REML, with and without random pairwise interactions in each year individually, and compared using LRTs. The purpose of including random pairwise interactions in a given year is twofold: (1) if they are not needed, this indicates no evidence of lack of fit in the interaction effect explanation, (2) if they are needed, the extra variance parameter acknowledges that there is additional variability due to pairwise interactions and incorporates this extra uncertainty into standard errors, without the need for many additional fixed terms (Brophy et al. 2017). To counteract the boundary space problem (Self and Liang 1987) when testing the inclusion of random effects in each year, the P-values of the LRTs were halved (Littell et al. 2006).

## Results

### SPaCE Data Overview

**Fig. 1:**
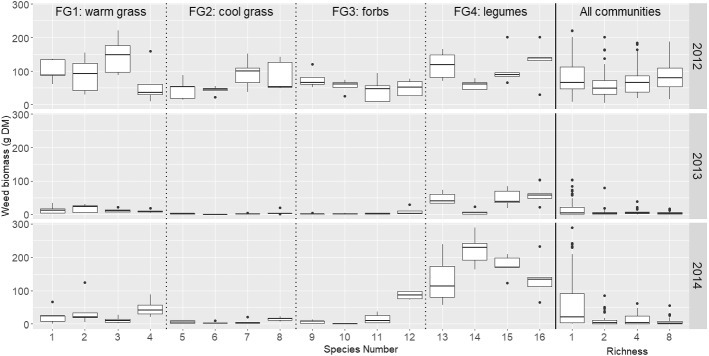
Total plot weed biomass (g DM in 1 m$^{2}$ plot) in 2012–2014 for each monoculture (1–16) and each level of richness (1, 2, 4 or 8 species). The 4 functional groups are separated by the dotted lines, while all communities are to the right of the solid line. The richness $=$ 1 boxplot describes all of the monocultures in each year

Weed biomass was similar for all levels of species richness in 2012 (Fig. 1; “All communities” panel). In 2013 and 2014, monocultures showed both a higher median and range in weed biomass than mixtures (Fig. 1; “All communities” panel), suggesting that diversity suppressed weed invasion over time. The medians and the variances of the weed biomass across the 16 monocultures varied in all years (Fig. 1). In 2013 and 2014, the mean and variance of weed biomass for legume monocultures (FG4) were considerably higher than all other types of communities (Fig. 1), confirming that homogeneity across all communities within each year is unlikely to be a valid assumption.

### Model Selected and its Interpretation

The results from steps 1 and 2 (listed in Sect. 2.4) are in supplementary materials S.3. In step 3, the best fixed effects model identified was the functional group model where spatial planting pattern interacted with the within functional group and between functional group interactions, and proportion of FG4 (legumes) interacted with additive species pairwise interactions (Table 2). In step 4, additional random pairwise interactions were needed in 2014 only (Table 2). The code to run these models is in supplementary materials S.4.

**Table 2:** Results from the tests conducted to determine the best linear predictor for the fixed effects (step 3) and tests for the inclusion of random pairwise interactions (step 4)

Model number	Model description	Test	LRT	df	*P*-value
*Fixed effects model comparisons*					
1	Average pairwise model				
2	Functional group model	1 v 2	62.8	27	<0.001
3	With functional group interactions * Spatial pattern	2 v 3	52.1	30	0.007
4	With additive species interactions * legume %	3 v 4	210.9	48	<0.001
*Random effects model comparisons*					
5	With random pairwise interactions in 2012	4 v 5	0	1	0.500
6	With random pairwise interactions in 2013	4 v 6	0.4	1	0.264
7	With random pairwise interactions in 2014	4 v 7	5.6	1	0.009

Likelihood ratio test statistics (LRT), degrees of freedom (df $=$ difference between the compared models) and P-values are shown. Model 1 includes block effects, identity effects for each species, and an average pairwise interaction term. Models 1–3 were fitted using maximum likelihood (ML). Model 4 was fitted by ML for comparisons of fixed effects and restricted maximum likelihood (REML) for comparisons of random effects. Models 5–7 were fitted using REML. The *P*-values for the random pairwise interactions variance components are halved due to the boundary space issue. The random pairwise interactions are tested for each year individually, and random pairwise interactions are included in the final model for all years that are significant

The final model contained the terms: identities $+$ within functional group by spatial pattern interaction $+$ between functional group by spatial pattern interactions $+$ additive species by legume percentage interactions $+$ random interactions for year 3 only. For year $k\, (k=1,\, 2,\, 3)$, block $l\, (l=1,\,\ldots ,\, 5)$, plot $m\, (1,\ldots ,\, 170)$, spatial pattern *n* (1 $=$ aggregated or 2 $=$ dispersed) and species *i*, *j*, the equation is:

5 $$\begin{aligned} y_{klmn}= & {} \sum _{i=1}^{16} \beta _{ik}P_{ikm}+\sum _{q=1}^4 \omega _{qqkn} \sum _{\tiny \begin{array}{l} i,j\in {FG}_{q} \\ i<j \\ \end{array}} {P_{ikm}P_{jkm}\, } \nonumber \\&+\, \sum _{1\le q<r\le 4} \omega _{qrkn} \sum _{i\in {FG}_{q}} \sum _{j\in {FG}_{r}} {P_{ikm}P_{jkm}} \, \nonumber \\&+\,\sum _{1\le i<j\le 16} {\left( \lambda _{ik}+\lambda _{jk} \right) P_{ikm}P_{jkm}} \sum _{i=13}^{16} P_{ikm} \nonumber \\&+\,\sum _{1\le i<j\le 16} {d_{ijk}P_{ikm}P_{jkm}X_{k}} +\alpha _{kl}+{\, \varepsilon }_{klmn} \end{aligned}$$

where ${\varvec{\varepsilon }}\sim N\left( {\varvec{0}},{\varvec{R}} \right) {\varvec{.}}~{\varvec{R}}$ contains different block diagonals for each functional group monoculture and another for mixtures (i.e. five repeated measures variance–covariance blocks across the 170 plots), $X_{k}=1$ if $k=3,$ and is 0 otherwise (since the random effects were only needed in year 3), $d_{ijk}\sim N(0,\sigma _{k}^{2})$ independent of ${\varvec{\varepsilon }}$. The $\alpha _{kl}$ parameters are the block effects and the $\beta _{ik}$ are the identity effects. The parameter $\omega _{qqkn}$ is the interaction between any pair of species from functional group *q*, for spatial pattern *n* in year *k*. The species in each functional group are: $\hbox {FG}_{1} = \{1,2,3,4\}$, $\hbox {FG}_{2} = \{ 5,6,7,8\}$ , $\hbox {FG}_{3}= \{ 9,10,11,12\}$ and $\hbox {FG}_{4} = \{ 13,14,15,16\}$. The parameter $\omega _{qrkn}$ is the interaction between any pair of species with one species each from functional group *q* and *r*, for spatial pattern *n* in year *k*. The $\lambda _{ik}$ terms are the fixed additive species interaction effects of species’ *i* and *j* in year *k*, and these are affected by legume percentage ($\sum _{i=13}^{16} P_{ikm} )$. The inclusion of random interaction effects in the final model means that in the third year of the experiment, there was additional variability due to the individual pairwise interactions that was not picked up by fixed interaction effects, and was incorporated into the fixed effects standard errors. The estimates of the fixed effects and variance components of the final model and a graphical assessment of model assumptions are included in supplementary materials S.5.

**Fig. 2:**
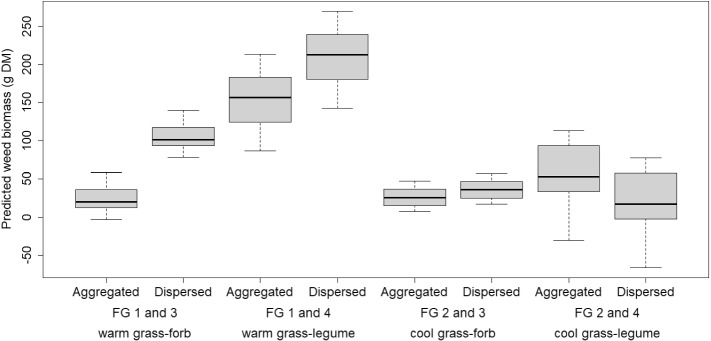
Predicted weed biomass (g DM in 1 m$^{2}$ plot) in 2012 in two-species centroid communities containing one species from either FG1 or FG2 and one species from either FG3 or FG4 split by spatial pattern (either aggregated or dispersed)

Species identity estimates in 2012 were all significantly higher than those in 2013 at the average block level ($P < 0.001$ in most cases; all $P< 0.02$), and in 2014 except for species 4, 11, 13 and 16. Two-species community predictions with both species in equal proportions (centroid communities) at the average block level were computed for all communities which contained one warm- or cool-season grass species and one forb or legume species; in 2012, the aggregated planting pattern generally yielded lower predictions than dispersed, except for communities mixing a species from FGs 2 and 4 (Fig. 2). This could be due to aggregation allowing subordinate species to establish more easily due to decreased interspecific interactions, leaving the community less susceptible to invasion at establishment (Yurkonis et al. 2012; Seahra et al. 2016). Model predictions showed increasing weeds as legume percentage increased (supplementary materials S.6).

## Simulation Studies

### Simulation Methods

We conducted simulation studies to evaluate the performance of the random effects approach to modeling pairwise interactions over multiple years; specifically, we tested the power of our approach to detect variation across pairwise interactions for a range of variance settings, and we assessed the ability of our model selection process to select the simulated model. The ‘design_a’ experimental design from the DImodels R package (Moral, Connolly, and Brophy 2021) was used. It contains $m=206\,$plots, a pool of nine species categorized according to three functional groups (each of size three), and plots of species richness 1, 2, 3, 4, 6, and 9. Each species was in monoculture in two plots; each pair of species appeared twice in a plot of richness two in equal proportion; and there were 48, 36, 24, and 8 plots of richness 3, 4, 6, and 9, respectively, with all species in equal proportion in each plot. Response values were simulated assuming a functional group model (described in Table 1) over $k=3$ experimental years with a compound symmetry variance–covariance structure. In addition to functional group interaction effects, extra variation due to pairwise interactions was included in the simulated responses. The simulated model was

6 $$\begin{aligned} y_{km}= & {} \sum _{i=1}^9 {\beta _{ik}P_{ikm}} +\sum _{q=1}^3 \omega _{qqk} \sum _{\tiny \begin{array}{l} i,j \in {FG}_{q} \\ i<j \\ \end{array}} {P_{ikm}P_{jkm}\, } \nonumber \\&+\, \sum _{1\le q<r\le 3} \omega _{qrk} \sum _{i\in {FG}_{q}} \sum _{j\in {FG}_{r}} {P_{ikm}P_{jkm}} \nonumber \\&+\,\sum _{1\le i<j\le 9} {d_{ijk}P_{ikm}P_{jkm}} +\varepsilon _{klm} \end{aligned}$$

where $P_{i}$ is the proportion of species *i* in the preceding year, $\beta _{ik}$ is the identity effect of species *i* in year *k*, $\omega _{qqk}$ is the interaction effect between any pair of species from functional group *q* in year *k*, and $\omega _{qrk}$ is the interaction effect between any pair of species from functional group *q* and functional group *r* in year *k*, with $\hbox {FG}_{1} = \{1,2,3\}$ , $\hbox {FG}_{2} = \{4,5,6\}$ and $\hbox {FG}_{3} = \{7,8,9\}$ . The identity effects values and the within and between functional group interaction values used in each year used for simulating the data are given in supplementary materials S.7. The random effects $d_{ijk}$ were assumed to be normally distributed in each year; the standard deviation values simulated (assumed the same in each year) were either 0, 500, 1000, 1500, 2000, or 2500. The residual errors $\varepsilon _{klm}$, also assumed normally distributed, were simulated assuming homogeneity across plots within each year, and there was a compound symmetry variance–covariance structure across years; the simulated standard deviation values were either 100, 200 or 300 for each year. The covariances of the residual errors were half of the variance values in each case. Each combination of random effects variance by error variance values gave rise to 18 sets of simulations, each of which contained 1000 datasets. The identity, average pairwise, functional group and full pairwise models were fitted to each of the simulated datasets with a compound symmetry variance–covariance structure, and compared to determine whether the functional group was determined as the best fixed effects model. When exploring whether random pairwise interactions were needed in the model, the functional group model was fitted without random pairwise interactions initially. Random pairwise interactions were added to the model in each year individually, and the number of times in which they were significant (using likelihood ratio tests) from the 1000 simulations was recorded. We also used the model selection process outlined in Sect. 2.4 to identify the best model for each simulated dataset. Some models did not initially converge because SAS fitted a highly negative covariance parameter estimate for the lin(1) parameter when no random pairwise interactions were present, or their variance was small compared to residual variance. When this happened, higher starting values of the variance were specified to help the model converge. The code to perform the simulation studies is in supplementary materials S.8.

### Simulation Results

The ability of the modeling approach to detect the random pairwise interactions in our simulation study varied depending on the combination of residual standard deviation and random pairwise interaction standard deviation (Table 3). When the random pairwise interactions standard deviation was set to 0, DI models found significant variation due to random pairwise interactions in one of the years on approximately 3% of occasions, indicating that there was approximately a 0.03 probability of a type 1 error (shown for each residual standard deviation value in Table 3). The power of the test to detect random pairwise interactions in all three years was always $> 0.9$, except for when the random pairwise interaction standard deviation was close to the residual error standard deviation (Table 3). For random pairwise interactions with standard deviation of 500, the power decreased from 0.992 to 0.571 to 0.076 for residual standard deviation 100, 200, and 300, respectively (although power to detect interactions in at least one year was much higher). Therefore, to be distinguishable from residual variance, random pairwise interactions must usually have sufficiently large variance in comparison with the residual variance.

**Table 3:** The number of simulations from 1000 that gave 0, 1, 2, or 3 significant yearly random pairwise interactions at the 0.05 significance level in each of three years for combinations of residual standard deviation by random pairwise interactions standard deviation

Residual standard deviation	Random pairwise interactions standard deviation	Number of significant yearly randomt pairwise interactions				(1) Type 1 error probability	(2) Power
		0	1	2	3		
100	0	966	33	1	0	0.034	–
100	500	0	0	8	992	–	0.992
100	1000	0	0	0	1000	–	1
100	1500	0	0	0	1000	–	1
100	2000	0	0	0	1000	–	1
100	2500	0	0	0	1000	–	1
200	0	976	24	0	0	0.024	–
200	500	1	49	379	571	–	0.571
200	1000	0	0	6	994	–	0.994
200	1500	0	0	0	1000	–	1
200	2000	0	0	1	999	–	0.999
200	2500	0	0	0	1000	–	1
300	0	971	28	1	0	0.029	–
300	500	211	409	304	76	–	0.076
300	1000	0	1	75	924	–	0.924
300	1500	0	0	2	998	–	0.998
300	2000	0	0	1	999	–	0.999
300	2500	0	0	0	1000	–	1

Two probabilities are shown: (1) for random pairwise interactions standard deviation $=$ 0, the probability of a type 1 error (i.e., the probably of falsely detecting a need for pairwise interactions in at least one year) and (2) for random pairwise interactions standard deviation $> 0$ the power to detect a need for random pairwise interactions in *all three years* (note that power to detect in at least one year would be higher)

When no random pairwise interactions were included, the functional group model was chosen as the best model in approximately 70% of cases, with the full pairwise model chosen in all other cases. When random pairwise interactions were included, the functional group model was chosen ahead of the identity and average pairwise models in all simulations, but the full pairwise model was preferable to the functional group model in almost all cases, regardless of residual error by pairwise interaction standard deviation combination.

## Discussion and Conclusions

Diversity-Interactions models are a valuable tool in BEF research: they can simultaneously assess the impact of individual species, their proportions and their interactions on ecosystem function (Kirwan et al. 2009). In this paper, we advance DI models using random effects to model numerous pairwise interactions in a multi-year setting. We showed that random effects component had a low probability of a type 1 error and strong power, although the power depended on the gap between random pairwise interaction standard deviation and the residual standard deviation. We applied the methods to a multi-year biodiversity experiment with 16 species and identified functional group interaction effects and planting spatial pattern effects.

The inclusion of the unusual random effects (indexed by pairwise species rather than by a plot-level factor) provides a novel and parsimonious way to model a large number of pairwise interactions over multiple years, extending the methods for a single year from Brophy et al. (2017). The ability of the modeling approach to detect the need for the random effects is determined by the relative size of the random effects standard deviation to the residual error standard deviation, but when relative size is sufficiently large, the model performs very well at detecting the need for the additional variance components. Our simulation studies also showed that when the random pairwise variance does not exist, the modeling approach has a low probability of a type 1 error and will detect that it is required less than 5% of the time. The analysis in Connolly et al. (2013) compared DI models to a reference model including a parameter for each distinct community and blocking structure or treatment providing a lack of fit test. The random effects approach here provides another lack of fit test, which focuses specifically on the interactions component of the DI model and does not require replication of distinct community compositions in the experimental design.

The simulation study showed mixed results in the ability of the approach to identify the best fixed effects model: when there was no random pairwise variation included, the functional group model was identified as best the majority of the time, but when random pairwise interaction variation was included, the full pairwise model was frequently identified as the best model. However, this result was not surprising, because as well as functional group interactions, there was additional variability due to the random pairwise interactions in the true underlying model and that variation was detected by the full pairwise model. In some experiments with large numbers of species and therefore a high number of pairwise interactions, it may not be possible to fit all of these pairwise interactions as fixed effects. If the fixed pairwise interactions can all be fitted, it might still not make biological sense to fit it due to a lack of interpretability with the high number of parameters. Including the additional variability as random pairwise interactions in our model allows us to account for the variability in a more parsimonious way as it uses one additional parameter for each year. However, this simulation study focused on one particular experimental design and set of fixed effects. Performing simulation studies in advance of designing/conducting similar experiments is recommended to help ensure there is sufficient power to observe any random pairwise interaction effects.

In our application to the SPaCE data, we identified varying abilities of species to resist weed invasion, with legumes proving particularly susceptible to invasion in monoculture. We also found that interaction effects were driven by functional group membership and that the functional group effects varied according to species spatial pattern. Thus, the spatial pattern effects depended on which species were in the mixture and could not be generalized to any mixture. Examining the raw data, when plots were grouped by spatial pattern within year, no apparent effect of spatial pattern treatment was observed in any of the years, except for a slightly inferior weed suppression in aggregated plots in 2013 and 2014 (supplementary materials S.9), highlighting the benefit of our approach over traditional ANOVA based methods for analyzing this type of data. Designing experiments intended for response surface analyses (Box and Draper 2007), such as our Diversity-Interactions model, can lead to more efficient designs (Cornell 2002). Previous studies have found that aggregation of plant species helped maintain diversity (Houseman 2014) and reduce weed invasion by allowing less competitive species to persist (Wassmuth et al. 2009). However, model predictions here suggested that aggregated plots were more prone to invasion outside the year of establishment, which is consistent with Yurkonis et al. (2012). We also identified a species-specific effect of the proportion of legumes in mixtures on the interaction effects: predicted weed biomass showed that dispersed spatial pattern communities in particular changed with increasing proportions of legumes. The results supported the argument that increasing the number of species with different functional traits has a positive impact on weed suppression (Hector et al. 2001; Pokorny et al. 2005).

We assumed that the random effects were normally distributed in the application of the approach to the SPaCE data and verified this was reasonable. The data in the simulation study were generated under the normality assumption for the random effects and future work could explore deviations from this. It is advisable to verify the random effects normality assumption in any application of the modeling approach. It may also be reasonable to assume a year-to-year covariance in the random pairwise interactions (nonzero covariances in the off-diagonals of the ${\varvec{M}}_{{\varvec{i,j}}}$ matrices described after Eq. (3)); code to illustrate implementing this is included in supplementary materials S.10.

The model applied to the SPaCE data adjusted interaction effects according to a spatial pattern treatment. At establishment, aggregated plots had higher mean total intraspecific interactions than dispersed plots, and the difference became larger with increasing richness because there was a higher chance of separation in the dispersed plots with more species (supplementary materials S.11). Future work could make bigger same-species clusters in the aggregated plots to increase the intraspecific interactions and further differentiate them from the dispersed plots (e.g., Seahra et al. 2016).

A log transformation of weed biomass is a possible alternative method of ensuring that the linear mixed model, as proposed here, provides a good fit to the data. However, a log transformation would reduce parameter interpretability. While allowing the block diagonal matrices of the ${\varvec{R}}$ matrix to vary according to plot characteristics introduces complexity to the model, it allows the parameters of the model to stay on the same scale as the response. Our approach respects the heterogenous variance pattern across the weed biomass responses while maintaining the analysis on the original scale of the data.

The SPaCE experimental design in this study partially confounded species richness and functional group richness: all two-species plots contained two functional groups (always a warm-season or cool-season grass with a legume or a forb), and all four-species and all eight-species plots contained four functional groups. The design was suitable for the aim of the study to investigate the interactions among different functional groups as they relate to a reconstructed grassland. However, there are implications of the restricted design space; for our estimated model, it was not reasonable to predict for two species plots with both species being from the same functional group, or for two grasses (warm and cool season) together, or a legume and a forb together. Generally, when predicting from DI models, consideration should be given to the representation around the simplex space in the study.

In conclusion, we have developed an advanced DI model incorporating spatial planting pattern treatment, heterogeneous residual variance, and a parsimonious description of high number of species interactions over multiple years. These models can be fitted using SAS and R providing techniques for analyzing multi-year BEF experiments with high number of species that do not rely on species richness as the sole driver of species mixing effects.

## Supplementary Information

Below is the link to the electronic supplementary material.

Supplementary file 1 (docx 258 KB)

Supplementary file 2 (csv 240 KB)

Supplementary file 3 (csv 216 KB)

Supplementary file 4 (csv 487 KB)
