# Supplementary material for: A Mixed Model for Assessing the Effect of Numerous Plant Species Interactions on Grassland Biodiversity and Ecosystem Function Relationships
Source: J Agric Biol Environ Stat. 2022 Sep 1;28(1):1–19. doi: 10.1007/s13253-022-00505-2 (PMC9908731; doi:10.1007/s13253-022-00505-2)
Supplement: Supplementary file 1 — (docx 258 KB) [file 13253_2022_505_MOESM1_ESM.docx]

**Supplementary material**

**S.1: Plot species spatial pattern arrangement examples**

| 1 | 1 | 1 | 1 | 1 | 1 | 1 | 1 |
| --- | --- | --- | --- | --- | --- | --- | --- |
| 1 | 1 | 1 | 1 | 1 | 1 | 1 | 1 |
| 1 | 1 | 1 | 1 | 1 | 1 | 1 | 1 |
| 1 | 1 | 1 | 1 | 1 | 1 | 1 | 1 |
| 1 | 1 | 1 | 1 | 1 | 1 | 1 | 1 |
| 1 | 1 | 1 | 1 | 1 | 1 | 1 | 1 |
| 1 | 1 | 1 | 1 | 1 | 1 | 1 | 1 |
| 1 | 1 | 1 | 1 | 1 | 1 | 1 | 1 |

(a)

(b) (c)

| 1 | 1 | 4 | 1 | 3 | 3 | 1 | 1 |
| --- | --- | --- | --- | --- | --- | --- | --- |
| 2 | 2 | 1 | 3 | 4 | 1 | 4 | 3 |
| 4 | 1 | 3 | 2 | 2 | 4 | 4 | 2 |
| 2 | 2 | 4 | 2 | 2 | 1 | 4 | 2 |
| 3 | 3 | 3 | 4 | 4 | 2 | 3 | 3 |
| 1 | 4 | 3 | 3 | 2 | 1 | 1 | 1 |
| 4 | 4 | 2 | 3 | 1 | 4 | 2 | 4 |
| 3 | 4 | 1 | 3 | 3 | 1 | 2 | 2 |

| 4 | 4 | 1 | 1 | 3 | 3 | 4 | 4 |
| --- | --- | --- | --- | --- | --- | --- | --- |
| 4 | 4 | 1 | 1 | 3 | 3 | 4 | 4 |
| 2 | 2 | 2 | 2 | 1 | 1 | 4 | 4 |
| 2 | 2 | 2 | 2 | 1 | 1 | 4 | 4 |
| 3 | 3 | 4 | 4 | 1 | 1 | 3 | 3 |
| 3 | 3 | 4 | 4 | 1 | 1 | 3 | 3 |
| 1 | 1 | 2 | 2 | 3 | 3 | 2 | 2 |
| 1 | 1 | 2 | 2 | 3 | 3 | 2 | 2 |

Figure 1: Hypothetical illustration of the planted species arrangement in the experimental plots. Each plot was 1 × 1 m and was divided into a grid of 8 × 8 cells at planting. A single individual was planted in each of the resulting 64 cells. Shown are examples of (a) a monoculture plot of species 1 where all cells were planted with individuals of the same species, (b) an aggregated plot where individuals of four species (species richness = 4, evenness = 1) were randomly assigned to and planted in groups of four cells, and (c) a dispersed plot where individuals of four species (species richness = 4, evenness = 1) were randomly assigned to and planted in individual cells.

**S.2: Tutorial style guide to fitting DI models in SAS and R**

**DI model coding in SAS for a simulated dataset with a covariance structure (different blocks for monocultures and mixtures) and random pairwise interactions over 3 experimental years.**

Data were simulated to have a functional group structure with random pairwise interactions where the $\boldsymbol{A}_{\boldsymbol{i}}$ matrices within the$\boldsymbol{R}$ matrix differed for monocultures and mixtures (monomix) in each year. The ‘design_a’ experimental design from the DImodels R package (de Andrade Moral, Connolly, and Brophy 2020) was generated with $m=206$plots, a pool of nine species categorized into three functional groups (each with three species), and plots containing 1, 2, 3, 4, 6, or 9 species. Each species was in two monoculture plots; each pair of species appeared in two 2-species plots in equal proportion; and there were 48, 36, 24, and 8 plots of 3, 4, 6, and 9 species respectively, with all species in equal proportion in each plot. Response values were simulated assuming a functional group model (Table 4, supplementary material S.7) over $k=3$ experimental years. In addition to functional group interaction effects, extra variation due to pairwise interactions was included in the simulated responses.

/*IMPORT THE DATASET*/

PROC IMPORT OUT=sim_data_FG_monomix

DATAFILE="'…/sim_data_FG_monomix.csv"

DBMS=CSV REPLACE;

GETNAMES=YES;

RUN;

/* CREATING A DATA SET NEEDED FOR THE RANDOM EFFECTS SPECIFICATION (LDATA) */

data re;

do i=1 to 36;

parm=1;

row=i;

col=i;

value=1;

output;

end;

drop i;

run;

/* Functional group model with random pairwise interactions and variance split by monocultures and mixtures */

proc mixed data=sim_data_FG_monomix method=reml;

class year plot monomix;

model y = P1*year P2*year P3*year P4*year P5*year P6*year P7*year P8*year P9*year PPwfg1*year PPwfg2*year PPwfg3*year PPbfg12*year PPbfg13*year PPbfg23*year / noint solution;

repeated year / subject=plot type=un group=monomix;

random Y1PP1-Y1PP36/ type=lin(1) ldata=re solution;

random Y2PP1-Y2PP36/ type=lin(1) ldata=re solution;

random Y3PP1-Y3PP36/ type=lin(1) ldata=re solution;

run;

- The class statement states which of the variables are categorical.
- The model statement specifies the response (y) and the explanatory variables, in this case a functional group model in which every term is crossed with year to obtain an estimate for each identity and diversity effect in each year.
- The repeated statement gives the variable that indexes the repeated measurements (year = 1, 2 ,3) and the subject in which the repeated measurements are recorded (plot = 1,...,206). Type specifies the covariance model used (in this case unstructured *i.e.*, within-subject errors for each pair of years have a unique covariance). Group specifies the groups that form the blocks of the$\boldsymbol{R}$ matrix. In this case, the blocks of the$\boldsymbol{R}$ matrix differed for monocultures and mixtures in each year, but this can take other forms.
- Each of the Y1PP1 - Y1PP36 random effects is a column containing the values of the 36 pairwise species interactions in year 1 at every plot in every year, meaning that in years 2 and 3 all of the Y1PP1 - Y1PP36 values are zero. Y2PP1 – Y2PP36 and Y3PP1 – Y3PP36 are similar for year 2 and year 3 respectively. The ldata re is an identity matrix with 36 rows. Type=lin(1) allows us to fit the unusual random effects which share a common variance when we specify ldata=re.

The code below shows how to specify the three years of random pairwise interactions in one statement, using type=lin(3), different ldata (re2) and a different ordering of the columns containing the values of the 36 pairwise interactions in each year. However, this specification does not allow the random effects to be fitted in individual years as easily.

/* CREATING A DATA SET NEEDED FOR THE SPECIFICATION OF RANDOM EFFECTS IN ONE STATEMENT */

data re2;

do i=1 to 36;

do j=1 to 3;

parm=j;

row=3*(i-1) +j;

col=3*(i-1)+j;

value=1;

output;

end;

end;

drop i j;

run;

/* Functional group model with random pairwise interactions and variance split by monocultures and mixtures */

proc mixed data=sim_data_FG_monomix method=reml;

class year plot monomix;

model y = P1*year P2*year P3*year P4*year P5*year P6*year P7*year P8*year P9*year P10*year PPwfg1*year PPwfg2*year PPwfg3*year PPbfg12*year PPbfg13*year PPbfg23*year / noint solution;

repeated year / subject=plot type=un group=monomix;

random Y1PP1 Y2PP1 Y3PP1 Y1PP2 Y2PP2 Y3PP2 Y1PP3 Y2PP3 Y3PP3 Y1PP4 Y2PP4 Y3PP4 Y1PP5 Y2PP5 Y3PP5 Y1PP6 Y2PP6 Y3PP6 Y1PP7 Y2PP7 Y3PP7 Y1PP8 Y2PP8 Y3PP8 Y1PP9 Y2PP9 Y3PP9 Y1PP10 Y2PP10 Y3PP10 Y1PP11 Y2PP11 Y3PP11 Y1PP12 Y2PP12 Y3PP12 Y1PP13 Y2PP13 Y3PP13 Y1PP14 Y2PP14 Y3PP14 Y1PP15 Y2PP15 Y3PP15 Y1PP16 Y2PP16 Y3PP16 Y1PP17 Y2PP17 Y3PP17 Y1PP18 Y2PP18 Y3PP18 Y1PP19 Y2PP19 Y3PP19 Y1PP20 Y2PP20 Y3PP20 Y1PP21 Y2PP21 Y3PP21 Y1PP22 Y2PP22 Y3PP22 Y1PP23 Y2PP23 Y3PP23 Y1PP24 Y2PP24 Y3PP24 Y1PP25 Y2PP25 Y3PP25 Y1PP26 Y2PP26 Y3PP26 Y1PP27 Y2PP27 Y3PP27 Y1PP28 Y2PP28 Y3PP28 Y1PP29 Y2PP29 Y3PP29 Y1PP30 Y2PP30 Y3PP30 Y1PP31 Y2PP31 Y3PP31 Y1PP32 Y2PP32 Y3PP32 Y1PP33 Y2PP33 Y3PP33 Y1PP34 Y2PP34 Y3PP34 Y1PP35 Y2PP35 Y3PP35 Y1PP36 Y2PP36 Y3PP36 / type=lin(3) ldata=re2 solution;

run;

**DI model fitting in SAS and R for a simulated data with an identity effect structure, simple covariance structure (no covariances among years) and random pairwise interactions over 3 experimental years.**

The following SAS code can be used to estimate the DI model for a simulated data set representing an experiment with 9 species, 206 plots and 3 years, totaling 618 observations. The code uses similar methods to that in the first part of supplement S.2 (DI model coding in SAS for a simulated dataset), where the methods are described in detail.

/*IMPORT THE DATASET*/

PROC IMPORT OUT=sim_data_R_SAS

DATAFILE="'/folders/myfolders/sim_data_R_SAS.csv"

DBMS=CSV REPLACE;

GETNAMES=YES;

RUN;

/* CREATING A DATA SET NEEDED FOR THE RANDOM EFFECTS SPECIFICATION*/

data re;

do i=1 to 36;

parm=1;

row=i;

col=i;

value=1;

output;

end;

drop i;

run;

/* Identity model with random pairwise interactions REML */

proc mixed data=sim_data_R_SAS method=reml;

class year plot;

model y = P1*year P2*year P3*year P4*year P5*year P6*year P7*year P8*year P9*year / noint solution;

repeated year / subject=plot type=vc;

random Y1PP1-Y1PP36/ type=lin(1) ldata=re solution;

random Y2PP1-Y2PP36/ type=lin(1) ldata=re solution;

random Y3PP1-Y3PP36/ type=lin(1) ldata=re solution;

run;

/* Identity model with random pairwise interactions ML */

proc mixed data=sim_data_R_SAS method=ml;

class year plot;

model y = P1*year P2*year P3*year P4*year P5*year P6*year P7*year P8*year P9*year / noint solution;

repeated year / subject=plot type=vc;

random Y1PP1-Y1PP36/ type=lin(1) ldata=re solution;

random Y2PP1-Y2PP36/ type=lin(1) ldata=re solution;

random Y3PP1-Y3PP36/ type=lin(1) ldata=re solution;

run;

The following R code can be used to estimate the DI model for a simulated data set (uploaded as Suppl. Material) representing an experiment with 9 species, 206 plots and 3 years, totaling 618 observations. We include only identity effects by year as fixed, and random pairwise interactions with a different variance per year as the linear predictor. Note that the open-source, free R packages lme4 and nlme cannot be used here, since their syntax does not allow for the specification of the random effects without a grouping factor. Hence, we write code that optimizes the profile (restricted) log-likelihood function for the variance components.

First, we read the data into R and import packages “optimx” (Nash and Varadhan, 2011) and “bbmle” (Bolker and R Development Core Team, 2016), which will be used to perform optimization:

sdata <- read.csv("sim_data_R_SAS.csv", h = T)

sdata$Year <- as.factor(sdata$Year)

sdata$Plot <- as.factor(sdata$Plot)

sdata$PlotYear <- with(sdata, Plot:Year)

sdata.x <- sdata

rownames(sdata.x) <- sdata.x$PlotYear

sdata <- sdata.x[levels(sdata$PlotYear),]

require(optimx)

require(bbmle)

We now code the X and Z matrices (see eq. 3 of the manuscript):

nPlots <- 216

nSpecies <- 9

nPairwise <- choose(nSpecies, 2)

fmlaX <- as.formula(paste("~ 0 + (", paste("P", 1:nSpecies,

sep = "", collapse = "+"),

"):Year"))

fmlaZ <- as.formula(paste("~ 0 + ((", paste("P", 1:nSpecies,

sep = "", collapse = "+"),

")^2):Year"))

X <- model.matrix(fmlaX, data = sdata)

Z <- model.matrix(fmlaZ, data = sdata)[,-c(1:(nSpecies*3))]

The Z matrix assumes non-standard form for mixed models, since there are no grouping factors. We may visualise it by executing

require(Matrix)

image(Matrix(Z))

We then write functions that generate the R, G, and V matrices, with V = ZGZ’ + R the marginal variance-covariance matrix of Y:

G <- function(sigma.vector, Z) {

nZ <- ncol(Z)/3

Gmat <- diag(rep(sigma.vector, nZ))

return(Gmat)

}

R <- function(sigmaR, y) {

n <- length(y)

Rmat <- diag(rep(sigmaR, n))

return(Rmat)

}

V <- function(sigma.vector, Z, y) {

Gmat <- G(sigma.vector[1:3], Z = Z)

Rmat <- R(sigma.vector[4], y = y)

Vmat <- Z%*%Gmat%*%t(Z) + Rmat

return(Vmat)

}

Given V, we may estimate the fixed effects using weighted least squares estimation, with the inverse of V as the matrix of weights, coded below:

beta.hat <- function(y, X, Vinv) {

XVX <- t(X)%*%Vinv%*%X

XVXinv <- solve(XVX)

XVy <- t(X)%*%Vinv%*%y

bh <- XVXinv%*%XVy

return(bh)

}

We then write the profile (restricted) log-likelihood function to be maximized to obtain the estimates for the variance components (note that we multiply the function by -1, because the algorithms used are minimizers). We include the argument REML to choose between ML (REML = FALSE) and REML (REML = TRUE) estimation. Also note that we apply exponentials to the variance components, to bound the parameter space (repar = TRUE, the default in the function):

profloglik <- function(sigma2G1, sigma2G2, sigma2G3, sigma2R,

y, X, Z, REML = FALSE, repar = TRUE) {

if(repar) {

sig.vec <- exp(c(sigma2G1, sigma2G2, sigma2G3, sigma2R))

} else {

sig.vec <- c(sigma2G1, sigma2G2, sigma2G3, sigma2R)

}

V.hat <- V(sigma.vector = sig.vec, Z = Z, y = y)

V.hat.inv <- solve(V.hat)

betas <- beta.hat(y = y, X = X, Vinv = V.hat.inv)

raw.res <- y - X%*%betas

llik <- -.5*(determinant(V.hat)$modulus + t(raw.res)%*%V.hat.inv%*%raw.res)

if(REML) llik <- llik -.5*determinant(t(X)%*%V.hat.inv%*%X)$modulus

minusloglik <- as.numeric(-llik)

return(minusloglik)

}

We use exp(10) as an initial value for all variance components and the bobyqa method, since we do not provide a gradient function, and because we have reparameterized the variance components, estimation is now unbounded. Note that depending on the initial values and the number of fixed effects that are included in the model, the algorithm may crash, and this is due to numerical instability that may require further investigation. We estimate the model using ML (fitML) and REML (fitREML):

init <- c(10,10,10,10)

names(init) <- c("sigma2G1","sigma2G2","sigma2G3","sigma2R")

fitML <- mle2(profloglik, start = as.list(init),

optimizer = "optimx", method = "bobyqa",

data = list(y = sdata$y,

X = X, Z = Z, REML = FALSE))

fitREML <- mle2(profloglik, start = as.list(init),

optimizer = "optimx", method = "bobyqa",

data = list(y = sdata$y,

X = X, Z = Z, REML = TRUE))

On a 4GB RAM with an Intel Core i5 processor machine, these model fitting procedures take less than 15 seconds each. We now may compile the estimates for the fixed effects, variance components, and predictions for the random effects for further inspection:

# Estimated variance components

sigML <- exp(fitML@coef)

sigREML <- exp(fitREML@coef)

# Estimated fixed effects

betasML <- beta.hat(y = sdata$y, X = X,

Vinv = solve(V(sigma.vector = sigML,

Z = Z, y = sdata$y)))

betasREML <- beta.hat(y = sdata$y, X = X,

Vinv = solve(V(sigma.vector = sigREML,

Z = Z, y = sdata$y)))

# Estimated marginal variance-covariance matrix (V)

G.hatML <- G(sigma.vector = sigML[1:3], Z = Z)

G.hatREML <- G(sigma.vector = sigREML[1:3], Z = Z)

V.hatML <- V(sigma.vector = sigML, Z = Z, y = sdata$y)

V.hatREML <- V(sigma.vector = sigREML, Z = Z, y = sdata$y)

# Predicted random effects

ranefsML <- G.hatML%*%t(Z) %*% solve(V.hatML) %*% (sdata$y - X%*%betasML)

ranefsREML <- G.hatREML%*%t(Z) %*% solve(V.hatREML) %*% (sdata$y - X%*%betasREML)

# Compilation of estimates and predictions

ranefs.rnames <- NULL

for(i in 1:36){

ranefs.rnames <- c(ranefs.rnames, paste("PP", i, ":year1", sep = ""),

paste("PP", i, ":year2", sep = ""),

paste("PP", i, ":year3", sep = ""))

}

ranefs <- data.frame(ranefsML, ranefsREML,

row.names = ranefs.rnames)

estimates <- data.frame("ML" = c(betasML, sigML),

"REML" = c(betasREML, sigREML),

row.names = c(paste("P", 1:9, ":year1", sep = ""),

paste("P", 1:9, ":year2", sep = ""),

paste("P", 1:9, ":year3", sep = ""),

"sigma2G1","sigma2G2","sigma2G3","sigma2R"))

round(ranefs, 2)

round(estimates, 2)

Object ranefs compiles the predicted random effects for each of the 36 pairwise interactions for each year. Object estimates compiles the estimated fixed effects (for this example only the identity effects by year).

**References**

Nash, J.C. and Varadhan, R. (2011) Unifying optimization algorithms to aid software system users: optimx for R. *Journal of Statistical Software* 43(9), 1-14.

Bolker, B. and R Development Core Team (2016) bbmle: Tools for general maximum likelihood estimation. R package version 1.0.18. <https://CRAN.R-project.org/package=bbml>

de Andrade Moral, R., Connolly, J., and Brophy, C. 2020. DImodels: Diversity-Interactions (DI) Models. R package version 1.0. https://CRAN.R-project.org/package=DImodels

**S.3: Results of steps 1 and 2 of the model fitting process applied to the SPaCE data.**

Following the steps in Section 2.4 of the main text, the additive species model was the best model identified in step 1 (which assumed an unstructured variance-covariance structure and homogeneity across plots within each year). In step 2, the best variance-covariance structure was unstructured block diagonals in the $\boldsymbol{R}$ matrix (i.e., a unique variance for each year and a unique covariance for each pair of years) but dependent on plot characteristics; characteristics were defined according to five categories: one for monoculture plots from each of the four functional groups, and another for mixture plots (Table 1 on next page).

(The results from steps 3 and 4 are given in the main text Section 3.2.)

Table 1. Details of step 2 of the model fitting process. Varying structures to account for repeated measures (CS=Compound Symmetry, AR(1)=Auto Regressive 1, UN=Unstructured) and the inclusion of variance-covariance blocks to account for inhomogeneous variance across plots are tested. These models were fitted using restricted maximum likelihood (REML). The fixed effects model selected in step 1 was the additive species model. Differences in AIC are from model 9, the best overall model which includes different blocks for each plant functional group (FG) in monoculture. The likelihood ratio test (LRT) statistic and p-value are also presented. Note that the structure in model 7 was used in Step 1.

| Model | Repeated measures | Variance-covariance block structure | No. parameters | $\Delta$ AIC | Model comparison | LRT stat | P-value |
| --- | --- | --- | --- | --- | --- | --- | --- |
| 1 | CS | Constant across all plots | 114 | 377.9 |  |  |  |
| 2 | CS | Different blocks for mixtures and monocultures | 116 | 378.4 | 2 v 1 | 3.5 | 0.174 |
| 3 | CS | Different blocks for mixtures and for each FG in monoculture | 122 | 338.8 | 3 v 1 | 55.1 | <0.001 |
| 4 | AR(1) | Constant across all plots | 114 | 377.2 |  |  |  |
| 5 | AR(1) | Different blocks for mixtures and monocultures | 116 | 378.7 | 5 v 4 | 2.5 | 0.287 |
| 6 | AR(1) | Different blocks for mixtures and for each FG in monoculture | 122 | 339.7 | 6 v 4 | 53.5 | <0.001 |
| 7 | UN | Constant across all plots | 118 | 84.0 |  |  |  |
| 8 | UN | Different blocks for mixtures and monocultures | 124 | 108.0 | 8 v 7 | 71 | <0.001 |
| 9 | UN | Different blocks for mixtures and for each FG in monoculture | 142 | 0 | 9 v 8 | 144 | <0.001 |

**S.4: Code from SAS to fit the DI models to the SPaCE data as in Section 3.2 of manuscript.**

/*IMPORT THE DATASET*/

PROC IMPORT OUT=space_data

DATAFILE="'/folders/myfolders/space_data.csv"

DBMS=CSV REPLACE;

GETNAMES=YES;

RUN;

/* CREATING A DATA SET NEEDED FOR THE RANDOM EFFECTS SPECIFICATION*/

data random_pairwise;

do i=1 to 120;

parm=1;

row=i;

col=i;

value=1;

output;

end;

drop i;

run;

The best model with basic covariance structure fitted in ML (step 1) was the additive species model.

/* Additive species model */

proc mixed data=space_data method=ml;

class block year plot;

model WeedBiomass = year year*p1 year*p2 year*p3 year*p4 year*p5 year*p6 year*p7 year*p8 year*p9 year*p10 year*p11 year*p12 year*p13 year*p14 year*p15 /* year*p16 */ year*block year*pq1 year*pq2 year*pq3 year*pq4 year*pq5 year*pq6 year*pq7 year*pq8 year*pq9 year*pq10 year*pq11 year*pq12 year*pq13 year*pq14 year*pq15 year*pq16 / noint solution;

repeated year / subject=plot type=un;

run;

Fit the best model with the different variance structures: monomix (different blocks for monocultures and mixtures) and comp2 (different blocks for monocultures in each FG and mixtures). Do this using CS, AR1 and UN covariance structures (step 2).

/* First using CS (Compound Symmetry) */

/* Additive species model */

proc mixed data=space_data method=reml;

class block year plot;

model WeedBiomass = year year*p1 year*p2 year*p3 year*p4 year*p5 year*p6 year*p7 year*p8 year*p9 year*p10 year*p11 year*p12 year*p13 year*p14 year*p15 /* year*p16 */ year*block year*pq1 year*pq2 year*pq3 year*pq4 year*pq5 year*pq6 year*pq7 year*pq8 year*pq9 year*pq10 year*pq11 year*pq12 year*pq13 year*pq14 year*pq15 year*pq16 / noint solution;

repeated year / subject=plot type=cs;

run;

/* Additive species model with monomix var structure */

/* Different blocks for monocultures and mixtures */

proc mixed data=space_data method=reml;

class block year plot monomix;

model WeedBiomass = year year*p1 year*p2 year*p3 year*p4 year*p5 year*p6 year*p7 year*p8 year*p9 year*p10 year*p11 year*p12 year*p13 year*p14 year*p15 /* year*p16 */ year*block year*pq1 year*pq2 year*pq3 year*pq4 year*pq5 year*pq6 year*pq7 year*pq8 year*pq9 year*pq10 year*pq11 year*pq12 year*pq13 year*pq14 year*pq15 year*pq16 / noint solution;

repeated year / subject=plot type=cs group=monomix;

run;

/* Additive species model with comp2 var structure */

/* Different blocks for monocultures in each FG and mixtures */

proc mixed data=space_data method=reml;

class block year plot comp2;

model WeedBiomass = year year*p1 year*p2 year*p3 year*p4 year*p5 year*p6 year*p7 year*p8 year*p9 year*p10 year*p11 year*p12 year*p13 year*p14 year*p15 /* year*p16 */ year*block year*pq1 year*pq2 year*pq3 year*pq4 year*pq5 year*pq6 year*pq7 year*pq8 year*pq9 year*pq10 year*pq11 year*pq12 year*pq13 year*pq14 year*pq15 year*pq16 / noint solution;

repeated year / subject=plot type=cs group=comp2;

run;

/* Next using AR(1), first-order auto-regressive */

/* Additive species model */

proc mixed data=space_data method=reml;

class block year plot;

model WeedBiomass = year year*p1 year*p2 year*p3 year*p4 year*p5 year*p6 year*p7 year*p8 year*p9 year*p10 year*p11 year*p12 year*p13 year*p14 year*p15 /* year*p16 */ year*block year*pq1 year*pq2 year*pq3 year*pq4 year*pq5 year*pq6 year*pq7 year*pq8 year*pq9 year*pq10 year*pq11 year*pq12 year*pq13 year*pq14 year*pq15 year*pq16 / noint solution;

repeated year / subject=plot type=ar(1);

run;

/* Additive species model with monomix var structure */

/* Different blocks for monocultures and mixtures */

proc mixed data=space_data method=reml;

class block year plot monomix;

model WeedBiomass = year year*p1 year*p2 year*p3 year*p4 year*p5 year*p6 year*p7 year*p8 year*p9 year*p10 year*p11 year*p12 year*p13 year*p14 year*p15 /* year*p16 */ year*block year*pq1 year*pq2 year*pq3 year*pq4 year*pq5 year*pq6 year*pq7 year*pq8 year*pq9 year*pq10 year*pq11 year*pq12 year*pq13 year*pq14 year*pq15 year*pq16 / noint solution;

repeated year / subject=plot type=ar(1) group=monomix;

run;

/* Additive species model with comp2 var structure */

/* Different blocks for monocultures in each FG and mixtures */

proc mixed data=space_data method=reml;

class block year plot comp2;

model WeedBiomass = year year*p1 year*p2 year*p3 year*p4 year*p5 year*p6 year*p7 year*p8 year*p9 year*p10 year*p11 year*p12 year*p13 year*p14 year*p15 /* year*p16 */ year*block year*pq1 year*pq2 year*pq3 year*pq4 year*pq5 year*pq6 year*pq7 year*pq8 year*pq9 year*pq10 year*pq11 year*pq12 year*pq13 year*pq14 year*pq15 year*pq16 / noint solution;

repeated year / subject=plot type=ar(1) group=comp2;

run;

/* Next using UN, unstructured */

/* Additive species model */

proc mixed data=space_data method=reml;

class block year plot;

model WeedBiomass = year year*p1 year*p2 year*p3 year*p4 year*p5 year*p6 year*p7 year*p8 year*p9 year*p10 year*p11 year*p12 year*p13 year*p14 year*p15 /* year*p16 */ year*block year*pq1 year*pq2 year*pq3 year*pq4 year*pq5 year*pq6 year*pq7 year*pq8 year*pq9 year*pq10 year*pq11 year*pq12 year*pq13 year*pq14 year*pq15 year*pq16 / noint solution;

repeated year / subject=plot type=un;

run;

/* Additive species model with monomix var structure */

/* Different blocks for monocultures and mixtures */

proc mixed data=space_data method=reml;

class block year plot monomix;

model WeedBiomass = year year*p1 year*p2 year*p3 year*p4 year*p5 year*p6 year*p7 year*p8 year*p9 year*p10 year*p11 year*p12 year*p13 year*p14 year*p15 /* year*p16 */ year*block year*pq1 year*pq2 year*pq3 year*pq4 year*pq5 year*pq6 year*pq7 year*pq8 year*pq9 year*pq10 year*pq11 year*pq12 year*pq13 year*pq14 year*pq15 year*pq16 / noint solution;

repeated year / subject=plot type=un group=monomix;

run;

/* Additive species model with comp2 var structure */

/* Different blocks for monocultures in each FG and mixtures */

proc mixed data=space_data method=reml;

class block year plot comp2;

model WeedBiomass = year year*p1 year*p2 year*p3 year*p4 year*p5 year*p6 year*p7 year*p8 year*p9 year*p10 year*p11 year*p12 year*p13 year*p14 year*p15 /* year*p16 */ year*block year*pq1 year*pq2 year*pq3 year*pq4 year*pq5 year*pq6 year*pq7 year*pq8 year*pq9 year*pq10 year*pq11 year*pq12 year*pq13 year*pq14 year*pq15 year*pq16 / noint solution;

repeated year / subject=plot type=un group=comp2;

run;

Fit fixed effects models with the best covariance structure (comp2) (step3).

/* Average pairwise model & comp2 */

proc mixed data=space_data method=ml;

class block year plot spatialpattern comp2;

model WeedBiomass = year year*p1 year*p2 year*p3 year*p4 year*p5 year*p6 year*p7 year*p8 year*p9 year*p10 year*p11 year*p12 year*p13 year*p14 year*p15 /* year*p16 */ year*block year*PPsum / noint solution;

repeated year / subject=plot type=un group=comp2;

run;

/* FG model & comp2 */

proc mixed data=space_data method=ml;

class block year plot spatialpattern comp2;

model WeedBiomass = year year*p1 year*p2 year*p3 year*p4 year*p5 year*p6 year*p7 year*p8 year*p9 year*p10 year*p11 year*p12 year*p13 year*p14 year*p15 /* year*p16 */ year*block year*PPwfg1 year*PPwfg2 year*PPwfg3 year*PPwfg4 year*PPbfg12 year*PPbfg13 year*PPbfg14 year*PPbfg23 year*PPbfg24 year*PPbfg34 / noint solution;

repeated year / subject=plot type=un group=comp2;

run;

/* FG model with functional group interactions * spatial pattern & comp2 */

proc mixed data=space_data method=ml;

class block year plot spatialpattern comp2;

model WeedBiomass = year year*p1 year*p2 year*p3 year*p4 year*p5 year*p6 year*p7 year*p8 year*p9 year*p10 year*p11 year*p12 year*p13 year*p14 year*p15 /* year*p16 */ year*block year*PPwfg1*spatialpattern year*PPwfg2*spatialpattern year*PPwfg3*spatialpattern year*PPwfg4*spatialpattern year*PPbfg12*spatialpattern year*PPbfg13*spatialpattern year*PPbfg14*spatialpattern year*PPbfg23*spatialpattern year*PPbfg24*spatialpattern year*PPbfg34*spatialpattern / noint solution;

repeated year / subject=plot type=un group=comp2;

run;

/* FG model with functional group interactions * spatial pattern & additive species interactions * legume % & comp2 */

proc mixed data=space_data method=ml;

class block year plot spatialpattern comp2;

model WeedBiomass = year year*p1 year*p2 year*p3 year*p4 year*p5 year*p6 year*p7 year*p8 year*p9 year*p10 year*p11 year*p12 year*p13 year*p14 year*p15 /* year*p16 */ year*block year*PPwfg1*spatialpattern year*PPwfg2*spatialpattern year*PPwfg3*spatialpattern year*PPwfg4*spatialpattern year*PPbfg12*spatialpattern year*PPbfg13*spatialpattern year*PPbfg14*spatialpattern year*PPbfg23*spatialpattern year*PPbfg24*spatialpattern year*PPbfg34*spatialpattern year*pq1*legumepc year*pq2*legumepc year*pq3*legumepc year*pq4*legumepc year*pq5*legumepc year*pq6*legumepc year*pq7*legumepc year*pq8*legumepc year*pq9*legumepc year*pq10*legumepc year*pq11*legumepc year*pq12*legumepc year*pq13*legumepc year*pq14*legumepc year*pq15*legumepc year*pq16*legumepc / noint solution;

repeated year / subject=plot type=un group=comp2;

run;

/* Include random effects in the best model */

/* First fit best model without random pairwise interactions using REML to allow comparisons */

/* FG model with DE*Space & legumepc*lambdas & comp2 */

proc mixed data=space_data method=reml;

class block year plot spatialpattern comp2;

model WeedBiomass = year year*p1 year*p2 year*p3 year*p4 year*p5 year*p6 year*p7 year*p8 year*p9 year*p10 year*p11 year*p12 year*p13 year*p14 year*p15 /* year*p16 */ year*block year*PPwfg1*spatialpattern year*PPwfg2*spatialpattern year*PPwfg3*spatialpattern year*PPwfg4*spatialpattern year*PPbfg12*spatialpattern year*PPbfg13*spatialpattern year*PPbfg14*spatialpattern year*PPbfg23*spatialpattern year*PPbfg24*spatialpattern year*PPbfg34*spatialpattern year*pq1*legumepc year*pq2*legumepc year*pq3*legumepc year*pq4*legumepc year*pq5*legumepc year*pq6*legumepc year*pq7*legumepc year*pq8*legumepc year*pq9*legumepc year*pq10*legumepc year*pq11*legumepc year*pq12*legumepc year*pq13*legumepc year*pq14*legumepc year*pq15*legumepc year*pq16*legumepc / noint solution;

repeated year / subject=plot type=un group=comp2;

run;

/* Include random effects in individual years */

/* FG model with DE*Space & legumepc*lambdas & comp2 & Y1 REs */

proc mixed data=space_data method=reml;

class block year plot spatialpattern comp2;

model WeedBiomass = year year*p1 year*p2 year*p3 year*p4 year*p5 year*p6 year*p7 year*p8 year*p9 year*p10 year*p11 year*p12 year*p13 year*p14 year*p15 /* year*p16 */ year*block year*PPwfg1*spatialpattern year*PPwfg2*spatialpattern year*PPwfg3*spatialpattern year*PPwfg4*spatialpattern year*PPbfg12*spatialpattern year*PPbfg13*spatialpattern year*PPbfg14*spatialpattern year*PPbfg23*spatialpattern year*PPbfg24*spatialpattern year*PPbfg34*spatialpattern year*pq1*legumepc year*pq2*legumepc year*pq3*legumepc year*pq4*legumepc year*pq5*legumepc year*pq6*legumepc year*pq7*legumepc year*pq8*legumepc year*pq9*legumepc year*pq10*legumepc year*pq11*legumepc year*pq12*legumepc year*pq13*legumepc year*pq14*legumepc year*pq15*legumepc year*pq16*legumepc / noint solution;

repeated year / subject=plot type=un group=comp2;

random Y1PP1-Y1PP120/ type=lin(1) ldata=random_pairwise solution;

run;

/* FG model with DE*Space & legumepc*lambdas & comp2 & Y2 REs */

proc mixed data=space_data method=reml;

class block year plot spatialpattern comp2;

model WeedBiomass = year year*p1 year*p2 year*p3 year*p4 year*p5 year*p6 year*p7 year*p8 year*p9 year*p10 year*p11 year*p12 year*p13 year*p14 year*p15 /* year*p16 */ year*block year*PPwfg1*spatialpattern year*PPwfg2*spatialpattern year*PPwfg3*spatialpattern year*PPwfg4*spatialpattern year*PPbfg12*spatialpattern year*PPbfg13*spatialpattern year*PPbfg14*spatialpattern year*PPbfg23*spatialpattern year*PPbfg24*spatialpattern year*PPbfg34*spatialpattern year*pq1*legumepc year*pq2*legumepc year*pq3*legumepc year*pq4*legumepc year*pq5*legumepc year*pq6*legumepc year*pq7*legumepc year*pq8*legumepc year*pq9*legumepc year*pq10*legumepc year*pq11*legumepc year*pq12*legumepc year*pq13*legumepc year*pq14*legumepc year*pq15*legumepc year*pq16*legumepc / noint solution;

repeated year / subject=plot type=un group=comp2;

random Y2PP1-Y2PP120/ type=lin(1) ldata=random_pairwise solution;

run;

/* FG model with DE*Space & legumepc*lambdas & comp2 & Y3 REs */

proc mixed data=space_data method=reml;

class block year plot spatialpattern comp2;

model WeedBiomass = year year*p1 year*p2 year*p3 year*p4 year*p5 year*p6 year*p7 year*p8 year*p9 year*p10 year*p11 year*p12 year*p13 year*p14 year*p15 /* year*p16 */ year*block year*PPwfg1*spatialpattern year*PPwfg2*spatialpattern year*PPwfg3*spatialpattern year*PPwfg4*spatialpattern year*PPbfg12*spatialpattern year*PPbfg13*spatialpattern year*PPbfg14*spatialpattern year*PPbfg23*spatialpattern year*PPbfg24*spatialpattern year*PPbfg34*spatialpattern year*pq1*legumepc year*pq2*legumepc year*pq3*legumepc year*pq4*legumepc year*pq5*legumepc year*pq6*legumepc year*pq7*legumepc year*pq8*legumepc year*pq9*legumepc year*pq10*legumepc year*pq11*legumepc year*pq12*legumepc year*pq13*legumepc year*pq14*legumepc year*pq15*legumepc year*pq16*legumepc / noint solution;

repeated year / subject=plot type=un group=comp2;

random Y3PP1-Y3PP120/ type=lin(1) ldata=random_pairwise solution;

run;

Run the best model using REML to assess assumptions and model parameter estimates. The estimate statement shows how to predict from the model for a four-species centroid community.

/* FG model with DE*Space & legumepc*lambdas & comp2 & Y3 REs */

proc mixed data=space_data method=reml plots=all;

class block year plot spatialpattern comp2;

model WeedBiomass = year year*p1 year*p2 year*p3 year*p4 year*p5 year*p6 year*p7 year*p8 year*p9 year*p10 year*p11 year*p12 year*p13 year*p14 year*p15 /* year*p16 */ year*block year*PPwfg1*spatialpattern year*PPwfg2*spatialpattern year*PPwfg3*spatialpattern year*PPwfg4*spatialpattern year*PPbfg12*spatialpattern year*PPbfg13*spatialpattern year*PPbfg14*spatialpattern year*PPbfg23*spatialpattern year*PPbfg24*spatialpattern year*PPbfg34*spatialpattern year*pq1*legumepc year*pq2*legumepc year*pq3*legumepc year*pq4*legumepc year*pq5*legumepc year*pq6*legumepc year*pq7*legumepc year*pq8*legumepc year*pq9*legumepc year*pq10*legumepc year*pq11*legumepc year*pq12*legumepc year*pq13*legumepc year*pq14*legumepc year*pq15*legumepc year*pq16*legumepc / noint solution;

repeated year / subject=plot type=un group=comp2;

random Y3PP1-Y3PP120/ type=lin(1) ldata=random_pairwise solution;

estimate ‘2014 disp species 1 5 9 15 avg block’ year 0 0 1 year*p1 0 0 0.25 year*p2 0 0 0 year*p3 0 0 0 year*p4 0 0 0 year*p5 0 0 0.25 year*p6 0 0 0 year*p7 0 0 0 year*p8 0 0 0 year*p9 0 0 0.25 year*p10 0 0 0 year*p11 0 0 0 year*p12 0 0 0 year*p13 0 0 0 year*p14 0 0 0 year*p15 0 0 0.25 year*block 0 0 0.2 0 0 0.2 0 0 0.2 0 0 0.2 0 0 0.2 year*PPwfg1*spatialpattern 0 0 0 0 0 0 0 0 0 year*PPwfg2*spatialpattern 0 0 0 0 0 0 0 0 0 year*PPwfg3*spatialpattern 0 0 0 0 0 0 0 0 0 year*PPwfg4*spatialpattern 0 0 0 0 0 0 0 0 0 year*PPbfg12*spatialpattern 0 0 0 0 0 0 0 0.0625 0 year*PPbfg13*spatialpattern 0 0 0 0 0 0 0 0.0625 0 year*PPbfg14*spatialpattern 0 0 0 0 0 0 0 0.0625 0 year*PPbfg23*spatialpattern 0 0 0 0 0 0 0 0.0625 0 year*PPbfg24*spatialpattern 0 0 0 0 0 0 0 0.0625 0 year*PPbfg34*spatialpattern 0 0 0 0 0 0 0 0.0625 0 year*pq1*legumepc 0 0 0.046875 year*pq2*legumepc 0 0 0 year*pq3*legumepc 0 0 0 year*pq4*legumepc 0 0 0 year*pq5*legumepc 0 0 0.046875 year*pq6*legumepc 0 0 0 year*pq7*legumepc 0 0 0 year*pq8*legumepc 0 0 0 year*pq9*legumepc 0 0 0.046875 year*pq10*legumepc 0 0 0 year*pq11*legumepc 0 0 0 year*pq12*legumepc 0 0 0 year*pq13*legumepc 0 0 0 year*pq14*legumepc 0 0 0 year*pq15*legumepc 0 0 0.046875 year*pq16*legumepc 0 0 0;

run;

**S.5: Estimates of the coefficients for the fixed effects, and the variance components of the final model. Model residual plots also included.**

Table 2. Estimates (Est) of the coefficients for the fixed effects of the final model with standard errors (SE) and P-values. The model was fitted with REML. Pleg is computed as $\sum_{i=13}^{16} P_{ikm}$ is the total legume proportion relevant to year $k$, plot $m$.

|  | Year | | | | | | | | |
| --- | --- | --- | --- | --- | --- | --- | --- | --- | --- |
|  | 2012 (*k* = 1) | | | 2013 (*k* = 2) | | | 2014 (*k* = 3) | | |
| Parameter | Est | SE | P-value | Est | SE | P-value | Est | SE | P-value |
| $\beta_{1k}$ | 68.0 | 19.26 | 0.0005 | 10.6 | 3.37 | 0.0018 | 8.9 | 8.89 | 0.3165 |
| $\beta_{2k}$ | 65.0 | 19.18 | 0.0008 | 12.2 | 3.50 | 0.0006 | 19.3 | 7.61 | 0.0117 |
| $\beta_{3k}$ | 127.4 | 21.38 | <.0001 | 11.8 | 4.25 | 0.0060 | 14.4 | 12.86 | 0.2646 |
| $\beta_{4k}$ | 32.7 | 19.53 | 0.0947 | 9.1 | 3.95 | 0.0224 | 50.0 | 11.68 | <.0001 |
| $\beta_{5k}$ | 26.3 | 14.09 | 0.0627 | 0.6 | 1.98 | 0.7452 | 4.8 | 3.65 | 0.1924 |
| $\beta_{6k}$ | 21.9 | 14.08 | 0.1200 | -0.3 | 1.93 | 0.8781 | 4.8 | 3.43 | 0.1635 |
| $\beta_{7k}$ | 72.9 | 14.71 | <.0001 | 0.6 | 2.11 | 0.7661 | 4.8 | 3.89 | 0.2185 |
| $\beta_{8k}$ | 62.9 | 14.20 | <.0001 | 5.4 | 2.06 | 0.0092 | 16.1 | 3.82 | <.0001 |
| $\beta_{9k}$ | 53.1 | 13.25 | <.0001 | 0.7 | 2.51 | 0.7898 | 6.9 | 5.07 | 0.1732 |
| $\beta_{10k}$ | 43.9 | 12.40 | 0.0005 | -0.5 | 2.48 | 0.8464 | 5.1 | 3.87 | 0.1899 |
| $\beta_{11k}$ | 24.0 | 12.84 | 0.0627 | 2.2 | 1.98 | 0.2702 | 23.3 | 4.00 | <.0001 |
| $\beta_{12k}$ | 29.6 | 12.39 | 0.0173 | 4.9 | 2.11 | 0.0205 | 85.5 | 4.30 | <.0001 |
| $\beta_{13k}$ | 96.3 | 19.32 | <.0001 | 47.9 | 9.98 | <.0001 | 130.3 | 24.62 | <.0001 |
| $\beta_{14k}$ | 39.9 | 19.33 | 0.0398 | 3.7 | 9.97 | 0.7114 | 226.7 | 24.39 | <.0001 |
| $\beta_{15k}$ | 85.2 | 19.31 | <.0001 | 51.4 | 9.92 | <.0001 | 169.9 | 24.19 | <.0001 |
| $\beta_{16k}$ | 107.7 | 19.27 | <.0001 | 56.5 | 9.97 | <.0001 | 135.5 | 24.64 | <.0001 |
| $\alpha_{k1}$ | 41.7 | 9.31 | <.0001 | 0.8 | 1.47 | 0.5720 | 2.4 | 2.57 | 0.3478 |
| $\alpha_{k2}$ | 32.9 | 9.35 | 0.0005 | 1.1 | 1.45 | 0.4330 | 5.7 | 2.63 | 0.0320 |
| $\alpha_{k3}$ | 21.3 | 9.45 | 0.0252 | 2.6 | 1.47 | 0.0817 | 2.6 | 2.68 | 0.3308 |
| $\alpha_{k4}$ | 7.7 | 9.50 | 0.4155 | 0.7 | 1.49 | 0.6554 | -7.9 | 2.73 | 0.0042 |
| $\alpha_{k5}$ | 0.0 | . | . | 0.0 | . | . | 0.0 | . | . |
| $\omega_{11k1}$ | 5494 | 2813.6 | 0.0518 | 403 | 444.8 | 0.3659 | 523 | 543.6 | 0.3367 |
| $\omega_{11k2}$ | 2639 | 1756.6 | 0.1341 | 201 | 582.3 | 0.7297 | 958 | 467.3 | 0.0413 |
| $\omega_{22k1}$ | -2948 | 1536.6 | 0.0560 | 247 | 362.2 | 0.4959 | -637 | 540.5 | 0.2394 |
| $\omega_{22k2}$ | -1974 | 1469.1 | 0.1802 | -25 | 201.8 | 0.9017 | -93 | 191.3 | 0.6260 |
| $\omega_{33k1}$ | 1109 | 1376.8 | 0.4214 | -43 | 46.7 | 0.3521 | 14 | 93.6 | 0.8813 |
| $\omega_{33k2}$ | 1979 | 1449.7 | 0.1733 | -110 | 63.5 | 0.0852 | 102 | 75.8 | 0.1798 |
| $\omega_{44k1}$ | -1724 | 2676.6 | 0.5199 | -23 | 1464.2 | 0.9874 | 919 | 1168.2 | 0.4322 |
| $\omega_{44k2}$ | -485 | 3133.5 | 0.8770 | -1526 | 2887.8 | 0.5977 | -6847 | 2763.0 | 0.0138 |
| $\omega_{12k1}$ | 39 | 377.6 | 0.9180 | -46 | 71.4 | 0.5190 | -123 | 84.6 | 0.1475 |
| $\omega_{12k2}$ | -890 | 374.5 | 0.0181 | 3 | 47.0 | 0.9528 | -13 | 70.4 | 0.8496 |
| $\omega_{13k1}$ | -209 | 183.8 | 0.2555 | -5 | 15.6 | 0.7624 | -22 | 38.9 | 0.5672 |
| $\omega_{13k2}$ | 115 | 72.2 | 0.1106 | 1 | 14.8 | 0.9611 | -77 | 36.1 | 0.0328 |
| $\omega_{14k1}$ | 2744 | 1139.7 | 0.0167 | 137 | 171.6 | 0.4268 | -1271 | 489.8 | 0.0099 |
| $\omega_{14k2}$ | 2967 | 1163.5 | 0.0113 | -336 | 448.1 | 0.4539 | -1802 | 562.0 | 0.0015 |
| $\omega_{23k1}$ | -145 | 95.4 | 0.1297 | 12 | 12.5 | 0.3425 | -62 | 26.9 | 0.0211 |
| $\omega_{23k2}$ | -105 | 80.8 | 0.1963 | -3 | 10.6 | 0.7427 | -88 | 23.1 | 0.0002 |
| $\omega_{24k1}$ | -6128 | 1910.7 | 0.0015 | -249 | 212.7 | 0.2421 | -536 | 149.1 | 0.0004 |
| $\omega_{24k2}$ | -6270 | 2040.3 | 0.0023 | -401 | 162.6 | 0.0143 | -57 | 161.7 | 0.7242 |
| $\omega_{34k1}$ | 1803 | 2028.0 | 0.3747 | 82 | 75.2 | 0.2743 | -24 | 108.2 | 0.8263 |
| $\omega_{34k2}$ | 2004 | 2089.5 | 0.3383 | 262 | 105.7 | 0.0136 | -152 | 160.0 | 0.3419 |
| $\lambda_{1k}$*PLeg | -5550 | 2348.9 | 0.0188 | -483 | 381.5 | 0.2062 | 1765 | 780.9 | 0.0245 |
| $\lambda_{2k}$*PLeg | -5710 | 2385.9 | 0.0173 | -199 | 390.8 | 0.6109 | 1439 | 631.1 | 0.0233 |
| $\lambda_{3k}$*PLeg | -5709 | 2440.7 | 0.0200 | -768 | 554.6 | 0.1674 | 721 | 661.5 | 0.2764 |
| $\lambda_{4k}$*PLeg | -5154 | 2369.2 | 0.0304 | -154 | 407.7 | 0.7065 | 1006 | 643.1 | 0.1189 |
| $\lambda_{5k}$*PLeg | 11254 | 3234.6 | 0.0006 | 447 | 283.3 | 0.1153 | 304 | 286.7 | 0.2894 |
| $\lambda_{6k}$*PLeg | 11572 | 3297.1 | 0.0005 | 596 | 343.9 | 0.0840 | 294 | 318.8 | 0.3565 |
| $\lambda_{7k}$*PLeg | 11624 | 3293.4 | 0.0005 | 437 | 302.1 | 0.1493 | 450 | 277.0 | 0.1050 |
| $\lambda_{8k}$*PLeg | 11661 | 3301.7 | 0.0005 | 636 | 295.4 | 0.0321 | 464 | 261.2 | 0.0770 |
| $\lambda_{9k}$*PLeg | -4072 | 3540.0 | 0.251 | -296 | 240.6 | 0.2201 | 137 | 305.9 | 0.6550 |
| $\lambda_{10k}$*PLeg | -3977 | 3625.2 | 0.2735 | 452 | 455.9 | 0.3227 | -80 | 297.3 | 0.7875 |
| $\lambda_{11k}$*PLeg | -3994 | 3627.2 | 0.2717 | -178 | 191.3 | 0.3519 | -292 | 257.4 | 0.2569 |
| $\lambda_{12k}$*PLeg | -4211 | 3579.1 | 0.2403 | -331 | 203.5 | 0.1049 | -85 | 283.3 | 0.7638 |
| $\lambda_{13k}$*PLeg | 637 | 934.0 | 0.4956 | 105 | 429.4 | 0.8073 | 1113 | 592.3 | 0.0612 |
| $\lambda_{14k}$*PLeg | 330 | 870.5 | 0.7048 | -18 | 162.9 | 0.9132 | -539 | 181.2 | 0.0032 |
| $\lambda_{15k}$*PLeg | 332 | 886.9 | 0.7081 | 113 | 404.9 | 0.7805 | 237 | 311.0 | 0.4465 |
| $\lambda_{16k}$*PLeg | 648 | 929.6 | 0.4865 | 1718 | 572.7 | 0.0029 | 3100 | 1109.1 | 0.0055 |

Table 3. Estimates of the variances and covariances (for the repeated measures on each plot) according to each type of plot (categorized by FG1 warm season grass, FG2 cool season grass, FG3 forbs and FG4 legume monocultures, and Mixture). The model was fitted with REML. The row and columns indicate the location in the 3 × 3 variance co-variance matrix. Variances are in bold. The variances of legume (FG4) monocultures is considerably higher than other groups in years 2 and 3, reflecting the variation patterns seen in Figure 1 in the main text.

|  |  |  | FG1 | FG2 | FG3 | FG4 | Mixture |
| --- | --- | --- | --- | --- | --- | --- | --- |
| Row | Column | Description | Estimate | | | | |
| 1 | 1 | Variance year 1 | **2311.4** | **915.2** | **747.8** | **1690.3** | **1811.2** |
| 2 | 1 | Covariance year 1 – 2 | 174.6 | -8.4 | 8.6 | -50.2 | 9.6 |
| 2 | 2 | Variance year 2 | **93.9** | **18.1** | **34.8** | **499.1** | **31.4** |
| 3 | 1 | Covariance year 1 – 3 | 435.5 | -68.0 | 56.4 | 124.9 | -269.9 |
| 3 | 2 | Covariance year 2 – 3 | 183.0 | 22.4 | -18.1 | 186.9 | 6.3 |
| 3 | 3 | Variance year 3 | **974.3** | **63.3** | **130.7** | **3062.9** | **80.2** |

Figure 3: Plots of conditional studentized residuals for weed biomass in the final selected model.

**S.6: Predicted weed biomass in in all four-species communities for both spatial patterns at varying levels of legume content.**


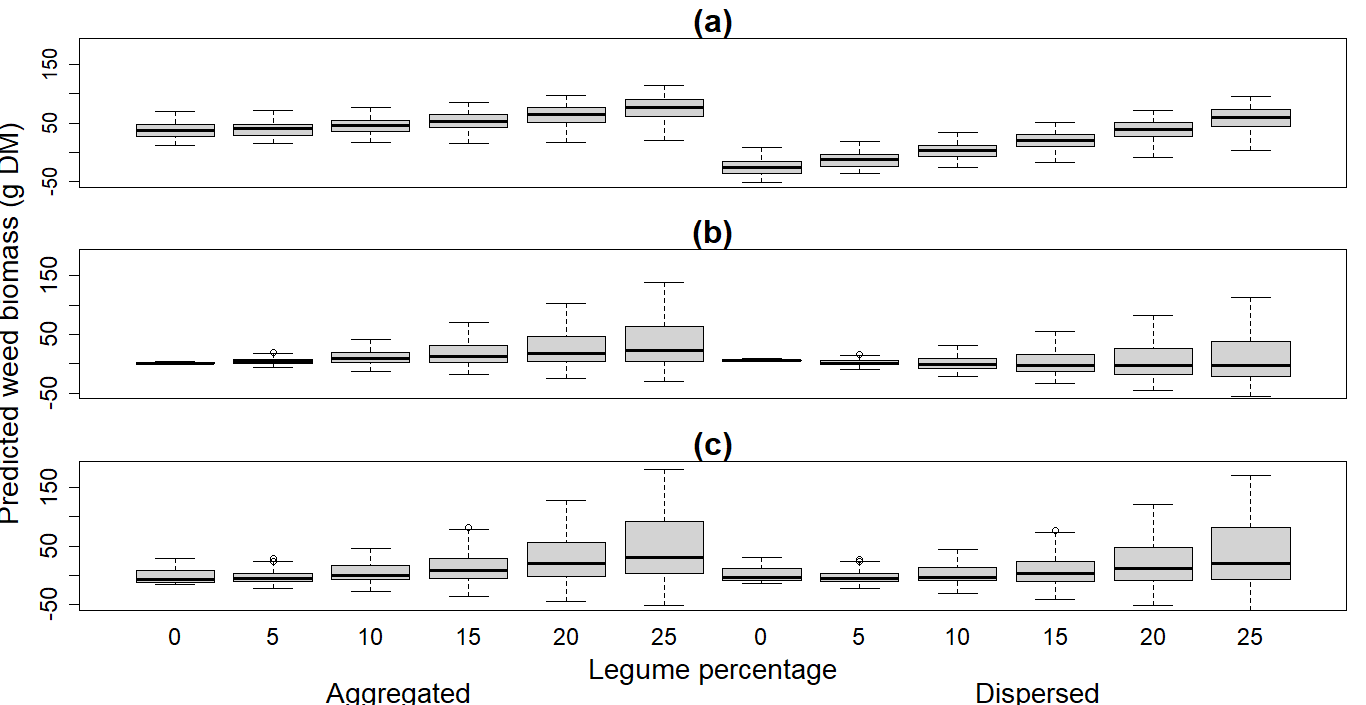


Figure 4. Predicted weed biomass (g DM in 1 m^2^ plot) in (a) 2012, (b) 2013, (c) 2014 in all four-species communities for both spatial patterns (aggregated and dispersed) containing one species from each functional group at the average block level, with legume percentage taking five values between 0% and 25% with the remaining percentage divided equally between the other three species. Some predictions were negative, but this was to be expected since there were a number of weed biomass observations close to and equal to zero in the SPaCE data. These negative predictions can be thought of as predicting that weeds were completely suppressed.

**S.7: Identity effects values and the within and between functional group interaction values in each year for the simulation study.**

Table 4. Values of the identity effects and the within and between functional group interactions in each year from the simulation study for model evaluation.

|  |  | Year |  |
| --- | --- | --- | --- |
| Effect | 1 | 2 | 3 |
| $\beta_{1}$ | 1700 | 2477 | 2103 |
| $\beta_{2}$ | 1472 | 2458 | 2411 |
| $\beta_{3}$ | 1512 | 2636 | 2601 |
| $\beta_{4}$ | 1650 | 2267 | 1604 |
| $\beta_{5}$ | 1440 | 1879 | 1753 |
| $\beta_{6}$ | 1343 | 1759 | 1792 |
| $\beta_{7}$ | 1709 | 1874 | 1637 |
| $\beta_{8}$ | 1699 | 2100 | 1752 |
| $\beta_{9}$ | 1584 | 1845 | 1748 |
| $\omega_{11}$ | 1400 | 1345 | 4592 |
| $\omega_{22}$ | 1753 | 1151 | 1954 |
| $\omega_{33}$ | 841 | 128 | 524 |
| $\omega_{12}$ | 2400 | 4345 | 4592 |
| $\omega_{13}$ | 1653 | 2151 | 1654 |
| $\omega_{23}$ | 4841 | 2028 | 3524 |

**S.8: R code to simulate the data and write it to csv files, SAS code to read in the data and fit the models necessary to determine the best fixed effects models and demonstrate detection of random pairwise interactions, and R code to find the best fixed effects models and test for inclusion of random pairwise interactions. The code to be changed to change the error standard deviation (100 in this case) and random pairwise interactions standard deviation (2500 in this case) are in bold.**

# Libraries used in this script

library(DImodels)

library(mvtnorm)

library(Matrix)

library(tidyverse)

# Set up proportions

# Simulate the data using the DImodels package experimental design design_a

data("design_a")

Jsim3a <- design_a

Jsim3a$plot <- 1:206

# Calculate PPwfg1, PPwfg2, PPwfg3 and PPbfg12, PPbfg13, PPbfg23

Jsim3a <- Jsim3a %>%

mutate(PPwfg1 = p1*p2 + p1*p3 + p2*p3,

PPwfg2 = p4*p5 + p4*p6 + p5*p6,

PPwfg3 = p7*p8 + p7*p9 + p8*p9,

PPbfg12 = p1*p4 + p1*p5 + p1*p6 + p2*p4 + p2*p5 + p2*p6 + p3*p4 + p3*p5 + p3*p6,

PPbfg13 = p1*p7 + p1*p8 + p1*p9 + p2*p7 + p2*p8 + p2*p9 + p3*p7 + p3*p8 + p3*p9,

PPbfg23 = p4*p7 + p4*p8 + p4*p9 + p5*p7 + p5*p8 + p5*p9 + p6*p7 + p6*p8 + p6*p9,)

# Repeat the experimental design over three years and include the identity effects in each year (Bi's)

all_years <- rbind.data.frame(

cbind('Year' = 1, Jsim3a),

cbind('Year' = 2, Jsim3a),

cbind('Year' = 3, Jsim3a))

all_years$Year <- as.factor(all_years$Year)

## X and Z matrices

nPlots <- 206

nSpecies <- 9

nPairwise <- choose(nSpecies, 2)

fmlaX <- as.formula(paste("~ 0 + (",

paste("p", 1:(nSpecies), sep = "", collapse = "+"),

"):Year + PPwfg1:Year + PPwfg2:Year + PPwfg3:Year + PPbfg12:Year + PPbfg13:Year + PPbfg23:Year"))

fmlaZ <- as.formula(paste("~ 0 + (", paste("p", 1:nSpecies,

sep = "", collapse = "+"),

")^2"))

X <- model.matrix(fmlaX, data = all_years)

X <- X[,!(colSums(X) == 0)]

PP1 <- model.matrix(fmlaZ, data = subset(all_years, Year == "1"))[,-c(1:nSpecies)]

PP2 <- model.matrix(fmlaZ, data = subset(all_years, Year == "2"))[,-c(1:nSpecies)]

PP3 <- model.matrix(fmlaZ, data = subset(all_years, Year == "3"))[,-c(1:nSpecies)]

zero.matrix <- matrix(rep(0, nPlots*nPairwise), ncol = nPairwise)

Z <- rbind(cbind(PP1, zero.matrix, zero.matrix),

cbind(zero.matrix, PP2, zero.matrix),

cbind(zero.matrix, zero.matrix, PP3))

Z

## Create Beta matrix

Beta <- matrix(c(1700, 2477, 2103, # P1 ID values in the 3 years

1472, 2458, 2411, # P2 ID values in the 3 years

1512, 2636, 2601, # P3 ID values in the 3 years

1650, 2267, 1604, # P4 ID values in the 3 years

1440, 1879, 1753, # P5 ID values in the 3 years

1343, 1759, 1792, # P6 ID values in the 3 years

1709, 1874, 1637, # P7 ID values in the 3 years

1699, 2100, 1752, # P8 ID values in the 3 years

1584, 1845, 1748, # P9 ID values in the 3 years

1400, 1345, 4592, # PPwfg1 values

1753, 1151, 1954, # PPwfg2 values

841, 128, 524, # PPwfg3 values

2400, 4345, 4592, # PPbfg12 values

1653, 2151, 1654, # PPbfg13 values

4841, 2028, 3524 # PPbfg23 values

), ncol = 1)

# Multiply X %*% Beta matrices

X_Beta <- X %*% Beta

# Create u and Epsilon matrices, find Y = x_Beta + Z_u + Epsilon, and add it to a dataframe

sim_func <- function(random_pair_int_sd, error_sd, X_B = X_Beta, Z_matrix = Z){

# Create u matrix

random_pair_int_var <- random_pair_int_sd^2

random_pair_int_cov <- 0

u <- as.vector(rmvnorm(n = 36, mean = c(0, 0, 0),

sigma = matrix(c(random_pair_int_var, random_pair_int_cov, random_pair_int_cov,

random_pair_int_cov, random_pair_int_var, random_pair_int_cov,

random_pair_int_cov, random_pair_int_cov, random_pair_int_var),

nrow = 3)))

error_var <- error_sd^2

error_cov <- error_var/2

# Now create the error terms matrix

Epsilon <- as.vector(rmvnorm(n = 206, mean = c(0, 0, 0),

sigma = matrix(c(error_var, error_cov, error_cov,

error_cov, error_var, error_cov,

error_cov, error_cov, error_var), nrow = 3)))

# Calculate x_Beta + Z%*%u + Epsilon

Y <- X_B + Z_matrix%*%u + Epsilon

Y

}

set.seed(534718)

all_sims_func <- function(random_pair_int_sd, error_sd){

sim_data <- 1000 %>%

rerun(cbind(Y = sim_func(random_pair_int_sd, error_sd), all_years)) %>%

bind_rows() %>%

mutate(Count = rep(1:1000, each = 618))

write.csv(sim_data,

paste0("file_path",

random_pair_int_sd, "_esd", error_sd, ".csv"),

row.names = F)

}

# Run this for all 6 rint sd values: seq(0, 2500, 500) and all 3 error sd values: c(100, 200, 300)

set.seed(534718)

all_sims_func(0,100)

map(seq(0, 2500, 500), function(i) {

map(c(100, 200, 300), function(j) {

all_sims_func(i, j)

})

})

/* IMPORT THE SIMULATED DATA */

**PROC** **IMPORT** OUT= RANDOMDI.data_**2500_100**_designa

DATAFILE="…\data_designa_rintsd**2500**_esd**100**.csv"

DBMS=CSV REPLACE;

GETNAMES=YES;

**RUN**;

/* ADD THE PLOT VARIABLE FROM 1 TO 206 IN EACH YEAR */

**data** RANDOMDI.data_**2500_100**_designa;

merge RANDOMDI.plot RANDOMDI.data_**2500_100**_designa;

**run**;

/* COMPUTE THE PIPJ PAIRWISE INTERACTION TERMS */

**data** RANDOMDI.data_**2500_100**_designa;

set RANDOMDI.data_**2500_100**_designa;

array p {**9**} p1-p9;

array pp {**36**};

l=**0**;

do i=**1** to **8**;

do j=(i+**1**) to **9**;

l=l+**1**;

pp{l}=p{i}*p{j};

end;

end;

drop i j l;

**run**;

/* SET UP THE DATASET SO THAT THE DI MODELS CAN BE FITTED */

**Data** RANDOMDI.data_**2500_100**_designa;

SET RANDOMDI.data_**2500_100**_designa;

/* ARRAY 1: CONTAINS THE SPECIES PROPORTIONS P1 TO P9 */

array A1 (**9**) P1-P9;

/* ARRAY 2: CONTAINS THE PAIRWISE SPECIES INTERACTIONS PP1-PP36

PPsum IS THE SUM OF PP1-PP36 */

k=**0**;

array A2 (**36**) PP1 - PP36;

PPsum=**0**;

do i=**1** to **8**;

do j=(i+**1**) to **9**;

k=k+**1**;

A2{k}=A1{i}*A1{j};

PPsum = PPsum + A2{k};

end;

end;

/* COMPUTE THE PQ VALUES FOR FITTING THE ADDITIVE SPECIES MODEL */

array A5 (**9**) PQ1 - PQ9;

do i=**1** to **9**;

A5{i}=A1{i}*(**1**-A1{i});

end;

drop i j k;

**run**;

/* SETTING UP SEPARATE PAIRWISE INTERACTIONS FOR EACH YEAR WITH ZEROS FOR OTHER YEARS*/

**data** RANDOMDI.data_**2500_100**_designa;

set RANDOMDI.data_**2500_100**_designa;

array PP {**36**} PP1 - PP36;

array PP_y1 {**36**} Y1PP1 - Y1PP36;

array PP_y2 {**36**} Y2PP1 - Y2PP36;

array PP_y3 {**36**} Y3PP1 - Y3PP36;

if year=**1**

then do i=**1** to **36**;

PP_y1{i}=PP{i};

PP_y2{i}=**0**;

PP_y3{i}=**0**;

end;

if year=**2**

then do i=**1** to **36**;

PP_y1{i}=**0**;

PP_y2{i}=PP{i};

PP_y3{i}=**0**;

end;

if year=**3**

then do i=**1** to **36**;

PP_y1{i}=**0**;

PP_y2{i}=**0**;

PP_y3{i}=PP{i};

end;

drop i;

**run**;

/* CREATING A DATA SET NEEDED FOR THE RANDOM EFFECTS SPECIFICATION*/

**data** RANDOMDI.re;

do i=**1** to **36**;

parm=**1**;

row=i;

col=i;

value=**1**;

output;

end;

drop i;

**run**;

/* CREATING A DATA SET NEEDED FOR THE RANDOM EFFECTS SPECIFICATION*/

**data** RANDOMDI.re_1000;

set RandomDI.re;

do Count = **1** to **1000**;

output;

end;

**run**;

**proc** **sort** data=RandomDI.re_1000;

by Count;

**run**;

/**************************************************************/

/* FIT DI MODELS TO THE DATA TO COMPARE FIXED EFFECTS MODELS */

/**************************************************************/

/* Set up dataset for output from models */

**data** RANDOMDI.fits_fix_**2500_100**_designa;

input Value Model $;

cards;

**run**;

**data** RANDOMDI.covs_fix_**2500_100**_designa;

input Value Model $;

cards;

**run**;

/* Identity Model */

**proc** **mixed** data=RANDOMDI.data_**2500_100**_designa method=ml;

where count <= **1000**;

by count;

class year plot;

model Y = P1*year P2*year P3*year P4*year P5*year P6*year P7*year P8*year P9*year / noint solution;

repeated year / subject=plot type=cs;

ods output FitStatistics=RANDOMDI.fits;

ods output CovParms=RANDOMDI.covs;

**run**;

**data** RANDOMDI.fits;

set RANDOMDI.fits;

Model='Iden';

**run**;

**data** RANDOMDI.covs;

set RANDOMDI.covs;

Model='Iden';

**run**;

**data** RANDOMDI.fits_fix_**2500_100**_designa;

set RANDOMDI.fits_fix_**2500_100**_designa RANDOMDI.fits;

**run**;

**data** RANDOMDI.covs_fix_**2500_100**_designa;

set RANDOMDI.covs_fix_**2500_100**_designa RANDOMDI.covs;

**run**;

/* Average Pairwise Model */

**proc** **mixed** data=RANDOMDI.data_**2500_100**_designa method=ml;

where count <= **1000**;

by count;

class year plot;

model Y = P1*year P2*year P3*year P4*year P5*year P6*year P7*year P8*year P9*year PPsum*year/ noint solution;

repeated year / subject=plot type=cs;

ods output FitStatistics=RANDOMDI.fits;

ods output CovParms=RANDOMDI.covs;

**run**;

**data** RANDOMDI.fits;

set RANDOMDI.fits;

Model='AvgP';

**run**;

**data** RANDOMDI.covs;

set RANDOMDI.covs;

Model='AvgP';

**run**;

**data** RANDOMDI.fits_fix_**2500_100**_designa;

set RANDOMDI.fits_fix_**2500_100**_designa RANDOMDI.fits;

**run**;

**data** RANDOMDI.covs_fix_**2500_100**_designa;

set RANDOMDI.covs_fix_**2500_100**_designa RANDOMDI.covs;

**run**;

/* Functional Group Model */

**proc** **mixed** data=RANDOMDI.data_**2500_100**_designa method=ml;

where count <= **1000**;

by count;

class year plot;

model Y = P1*year P2*year P3*year P4*year P5*year P6*year P7*year P8*year P9*year

PPwfg1*year PPwfg2*year PPwfg3*year PPbfg12*year PPbfg13*year PPbfg23*year / noint solution;

repeated year / subject=plot type=cs;

ods output FitStatistics=RANDOMDI.fits;

ods output CovParms=RANDOMDI.covs;

**run**;

**data** RANDOMDI.fits;

set RANDOMDI.fits;

Model='FuGr';

**run**;

**data** RANDOMDI.covs;

set RANDOMDI.covs;

Model='FuGr';

**run**;

**data** RANDOMDI.fits_fix_**2500_100**_designa;

set RANDOMDI.fits_fix_**2500_100**_designa RANDOMDI.fits;

**run**;

**data** RANDOMDI.covs_fix_**2500_100**_designa;

set RANDOMDI.covs_fix_**2500_100**_designa RANDOMDI.covs;

**run**;

/* Additive Species Model */

**proc** **mixed** data=RANDOMDI.data_**2500_100**_designa method=ml;

where count <= **1000**;

by count;

class year plot;

model Y = P1*year P2*year P3*year P4*year P5*year P6*year P7*year P8*year P9*year

year*pq1 year*pq2 year*pq3 year*pq4 year*pq5 year*pq6 year*pq7 year*pq8 year*pq9 / noint solution;

repeated year / subject=plot type=cs;

ods output FitStatistics=RANDOMDI.fits;

ods output CovParms=RANDOMDI.covs;

**run**;

**data** RANDOMDI.fits;

set RANDOMDI.fits;

Model='AdSp';

**run**;

**data** RANDOMDI.covs;

set RANDOMDI.covs;

Model='AdSp';

**run**;

**data** RANDOMDI.fits_fix_**2500_100**_designa;

set RANDOMDI.fits_fix_**2500_100**_designa RANDOMDI.fits;

**run**;

**data** RANDOMDI.covs_fix_**2500_100**_designa;

set RANDOMDI.covs_fix_**2500_100**_designa RANDOMDI.covs;

**run**;

/* Full Pairwise Model */

**proc** **mixed** data=RANDOMDI.data_**2500_100**_designa method=ml;

where count <= **1000**;

by count;

class year plot;

model Y = P1*year P2*year P3*year P4*year P5*year P6*year P7*year P8*year P9*year

year*pp1 year*pp2 year*pp3 year*pp4 year*pp5 year*pp6 year*pp7 year*pp8 year*pp9 year*pp10

year*pp11 year*pp12 year*pp13 year*pp14 year*pp15 year*pp16 year*pp17 year*pp18 year*pp19 year*pp20

year*pp21 year*pp22 year*pp23 year*pp24 year*pp25 year*pp26 year*pp27 year*pp28 year*pp29 year*pp30

year*pp31 year*pp32 year*pp33 year*pp34 year*pp35 year*pp36 / noint solution;

repeated year / subject=plot type=cs;

ods output FitStatistics=RANDOMDI.fits;

ods output CovParms=RANDOMDI.covs;

**run**;

**data** RANDOMDI.fits;

set RANDOMDI.fits;

Model='Full';

**run**;

**data** RANDOMDI.covs;

set RANDOMDI.covs;

Model='Full';

**run**;

**data** RANDOMDI.fits_fix_**2500_100**_designa;

set RANDOMDI.fits_fix_**2500_100**_designa RANDOMDI.fits;

**run**;

**data** RANDOMDI.covs_fix_**2500_100**_designa;

set RANDOMDI.covs_fix_**2500_100**_designa RANDOMDI.covs;

**run**;

/* Export the fits and covs csv files */

**proc** **export**

data=RANDOMDI.fits_fix_**2500_100**_designa

outfile='file_path\fits_fix_**2500_100**_designa.csv'

dbms=csv REPLACE;

**run**;

**proc** **export**

data=RANDOMDI.covs_fix_**2500_100**_designa

outfile='file_path \covs_fix_**2500_100**_designa.csv'

dbms=csv REPLACE;

**run**;

/**************************************************************/

/* FIT DI MODELS TO THE DATA TO TEST FOR RANDOM PAIRWISE INTS */

/**************************************************************/

/* Set up datasets to store output from models */

/* Want to store the log likelihood and AIC values */

**data** RANDOMDI.fits_**2500_100**;

input Value Model $;

cards;

**run**;

/* Want to store the covariance and lin(1) (variance of the random pairwise interactions) values */

**data** RANDOMDI.covs_**2500_100**;

input Value Model $;

cards;

**run**;

/* Functional Group Model without RE */

**proc** **mixed** data=RANDOMDI.data_**2500_100**_designa method=reml;

where count <= **1000**;

by count;

class year plot;

model Y = P1*year P2*year P3*year P4*year P5*year P6*year P7*year P8*year

P9*year PPwfg1*year PPwfg2*year PPwfg3*year PPbfg12*year PPbfg13*year PPbfg23*year/ noint solution;

repeated year / subject=plot type=cs;

ods output FitStatistics=RANDOMDI.fits;

ods output CovParms=RANDOMDI.covs;

**run**;

**data** RANDOMDI.fits;

set RANDOMDI.fits;

Model='FGWO';

**run**;

**data** RANDOMDI.covs;

set RANDOMDI.covs;

Model='FGWO';

**run**;

**data** RANDOMDI.fits_**2500_100**;

set RANDOMDI.fits_**2500_100** RANDOMDI.fits;

**run**;

**data** RANDOMDI.covs_**2500_100**;

set RANDOMDI.covs_**2500_100** RANDOMDI.covs;

**run**;

/* Functional Group Model with REs in Y1 */

**proc** **mixed** data=RANDOMDI.data_**2500_100**_designa method=reml noprofile scoring = **10** maxiter = **100**;

where count <= **1000**;

by count;

class year plot;

model Y = P1*year P2*year P3*year P4*year P5*year P6*year P7*year P8*year

P9*year PPwfg1*year PPwfg2*year PPwfg3*year PPbfg12*year PPbfg13*year PPbfg23*year/ noint solution;

repeated year / subject=plot type=cs;

parms (**6250000**) (**10000**) (**5000**) / nobound;

random Y1PP1-Y1PP36 / type=lin(**1**) ldata=RANDOMDI.re_1000 solution;

ods output FitStatistics=RANDOMDI.fits;

ods output CovParms=RANDOMDI.covs;

**run**;

**data** RANDOMDI.fits;

set RANDOMDI.fits;

Model='FGY1';

**run**;

**data** RANDOMDI.covs;

set RANDOMDI.covs;

Model='FGY1';

**run**;

**data** RANDOMDI.fits_**2500_100**;

set RANDOMDI.fits_**2500_100** RANDOMDI.fits;

**run**;

**data** RANDOMDI.covs_**2500_100**;

set RANDOMDI.covs_**2500_100** RANDOMDI.covs;

**run**;

/* Functional Group Model with REs in Y2 */

**proc** **mixed** data=RANDOMDI.data_**2500_100**_designa method=reml noprofile scoring = **10** maxiter = **100**;

where count <= **1000**;

by count;

class year plot;

model Y = P1*year P2*year P3*year P4*year P5*year P6*year P7*year P8*year

P9*year PPwfg1*year PPwfg2*year PPwfg3*year PPbfg12*year PPbfg13*year PPbfg23*year/ noint solution;

repeated year / subject=plot type=cs;

parms (**6250000**) (**10000**) (**5000**) / nobound;

random Y2PP1-Y2PP36 / type=lin(**1**) ldata=RANDOMDI.re_1000 solution;

ods output FitStatistics=RANDOMDI.fits;

ods output CovParms=RANDOMDI.covs;

**run**;

**data** RANDOMDI.fits;

set RANDOMDI.fits;

Model='FGY2';

**run**;

**data** RANDOMDI.covs;

set RANDOMDI.covs;

Model='FGY2';

**run**;

**data** RANDOMDI.fits_**2500_100**;

set RANDOMDI.fits_**2500_100** RANDOMDI.fits;

**run**;

**data** RANDOMDI.covs_**2500_100**;

set RANDOMDI.covs_**2500_100** RANDOMDI.covs;

**run**;

/* Functional Group Model with REs in Y3 */

**proc** **mixed** data=RANDOMDI.data_**2500_100**_designa method=reml noprofile scoring = **10** maxiter = **100**;

where count <= **1000**;

by count;

class year plot;

model Y = P1*year P2*year P3*year P4*year P5*year P6*year P7*year P8*year

P9*year PPwfg1*year PPwfg2*year PPwfg3*year PPbfg12*year PPbfg13*year PPbfg23*year/ noint solution;

repeated year / subject=plot type=cs;

parms (**6250000**) (**10000**) (**5000**) / nobound;

random Y3PP1-Y3PP36 / type=lin(**1**) ldata=RANDOMDI.re_1000 solution;

ods output FitStatistics=RANDOMDI.fits;

ods output CovParms=RANDOMDI.covs;

**run**;

**data** RANDOMDI.fits;

set RANDOMDI.fits;

Model='FGY3';

**run**;

**data** RANDOMDI.covs;

set RANDOMDI.covs;

Model='FGY3';

**run**;

**data** RANDOMDI.fits_**2500_100**;

set RANDOMDI.fits_**2500_100** RANDOMDI.fits;

**run**;

**data** RANDOMDI.covs_**2500_100**;

set RANDOMDI.covs_**2500_100** RANDOMDI.covs;

**run**;

/* Export the fits and cov values to csv files */

**proc** **export**

data=RANDOMDI.fits_**2500_100**

outfile='file_path/fits_**2500_100**.csv'

dbms=csv REPLACE;

**run**;

**proc** **export**

data=RANDOMDI.covs_**2500_100**

outfile='file_path/covs_**2500_100**.csv'

dbms=csv REPLACE;

**run**;

library(tidyverse)

# Run the tests to find the best fixed effects models

fits_fix_**2500_100**_designa <- read.csv("fits_fix_**2500_100**_designa.csv")

############################################################

# Write a function to see if the average pairwise is better than the identity

id_avg_func <- function(sim_stats){

best_id_avg_model <- vector(length=1000)

for(i in 1:1000){

if(pchisq(filter(sim_stats, Count==i, Model=="Iden")[1,1] - filter(sim_stats, Count==i, Model=="AvgP")[1,1],

df=3, lower.tail=FALSE) < 0.05){best_id_avg_model[i] <- "AvgP"}

else{best_id_avg_model[i] <- "Iden"}

}

return(best_id_avg_model)}

avg_fg_func <- function(sim_stats){

best_avg_fg_model <- vector(length=1000)

for(i in 1:1000){

if(pchisq(filter(sim_stats, Count==i, Model=="AvgP")[1,1] - filter(sim_stats, Count==i, Model=="FuGr")[1,1],

df=15, lower.tail=FALSE) < 0.05){best_avg_fg_model[i] <- "FuGr"}

else{best_avg_fg_model[i] <- "AvgP"}

}

return(best_avg_fg_model)}

# 90 degrees of freedom for 36*3 pairwise interactions minus 6*3 FG interactions (multiplied for 3 years)

fg_full_func <- function(sim_stats){

best_fg_full_model <- vector(length=1000)

for(i in 1:1000){

if(pchisq(filter(sim_stats, Count==i, Model=="FuGr")[1,1] - filter(sim_stats, Count==i, Model=="Full")[1,1],

df=90, lower.tail=FALSE) < 0.05){best_fg_full_model[i] <- "Full"}

else{best_fg_full_model[i] <- "FuGr"}

}

return(best_fg_full_model)}

# Run the functions for the fits_fix_2500_100_designa data

fits_**2500_100**_best_id_avg_model <- id_avg_func(fits_fix_**2500_100**_designa)

fits_**2500_100**_best_avg_fg_model <- avg_fg_func(fits_fix_**2500_100**_designa)

fits_**2500_100**_best_fg_full_model <- fg_full_func(fits_fix_**2500_100**_designa)

# Use table to see how many times each model is best in each of the 1000 cases

table(fits_**2500_100**_best_id_avg_model)

table(fits_**2500_100**_best_avg_fg_model)

table(fits_**2500_100**_best_fg_full_model)

# Run the tests to see whether random pairwise interactions are needed

sim_**2500_100** <- read.csv("file_path/fits_**2500_100**.csv")

best_RE_model_func_year1 <- function(sim_stats){

best_RE_model <- vector(length=1000)

for(i in 1:1000){

if(is.na(filter(sim_stats, Count==i, Model=="FGWO")[1,1] - filter(sim_stats, Count==i, Model=="FGY1")[1,1])){

best_RE_model[i] <- "NA"}

else if(pchisq(filter(sim_stats, Count==i, Model=="FGWO")[1,1] - filter(sim_stats, Count==i, Model=="FGY1")[1,1],

df=1, lower.tail=FALSE) < 0.025){best_RE_model[i] <- "FGY1"}

else{best_RE_model[i] <- "FGWO"}

}

return(best_RE_model)}

best_RE_model_func_year2 <- function(sim_stats){

best_RE_model <- vector(length=1000)

for(i in 1:1000){

if(is.na(filter(sim_stats, Count==i, Model=="FGWO")[1,1] - filter(sim_stats, Count==i, Model=="FGY2")[1,1])){

best_RE_model[i] <- "NA"}

else if(pchisq(filter(sim_stats, Count==i, Model=="FGWO")[1,1] - filter(sim_stats, Count==i, Model=="FGY2")[1,1],

df=1, lower.tail=FALSE) < 0.025){best_RE_model[i] <- "FGY2"}

else{best_RE_model[i] <- "FGWO"}

}

return(best_RE_model)}

best_RE_model_func_year3 <- function(sim_stats){

best_RE_model <- vector(length=1000)

for(i in 1:1000){

if(is.na(filter(sim_stats, Count==i, Model=="FGWO")[1,1] - filter(sim_stats, Count==i, Model=="FGY3")[1,1])){

best_RE_model[i] <- "NA"}

else if(pchisq(filter(sim_stats, Count==i, Model=="FGWO")[1,1] - filter(sim_stats, Count==i, Model=="FGY3")[1,1],

df=1, lower.tail=FALSE) < 0.025){best_RE_model[i] <- "FGY3"}

else{best_RE_model[i] <- "FGWO"}

}

return(best_RE_model)}

sim_**2500_100**_best_RE_model_year1 <- best_RE_model_func_year1(sim_**2500_100**)

sim_**2500_100**_best_RE_model_year2 <- best_RE_model_func_year2(sim_**2500_100**)

sim_**2500_100**_best_RE_model_year3 <- best_RE_model_func_year3(sim_**2500_100**)

**S.9: Weed biomass in aggregated and dispersed mixture plots in each year.**


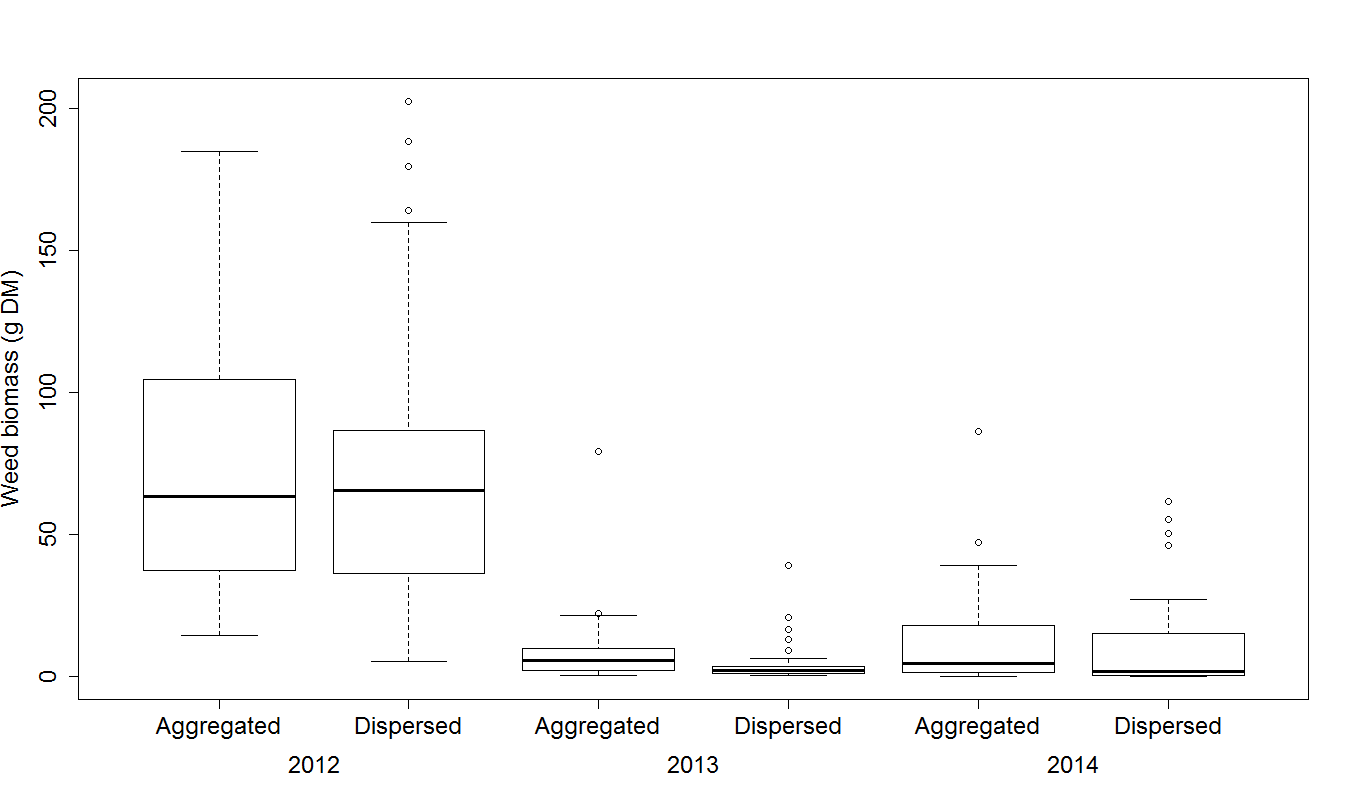


Figure 5. Box plots of weed biomass (total g DM /1 m^2^ plot) removed from aggregated and dispersed mixture plots in each experimental year.

**S.10: SAS code to fit correlated random pairwise interactions (to data from the simulation study)**

/* CREATING DATA NEEDED FOR THE SPECIFICATION OF CORRELATED RANDOM EFFECTS IN ONE STATEMENT */

**data** re3;

do i=**1** to **45**;

do j=**1** to **3**;

parm=j;

row=**3***(i-**1**)+j;

col=**3***(i-**1**)+j;

value=**1**;

output;

end;

end;

drop i j;

do i=**1** to **45**;

parm=**4**;

row=**3***(i-**1**)+**1**;

col=**3***(i-**1**)+**2**;

value=**1**;

output;

parm=**4**;

row=**3***(i-**1**)+**2**;

col=**3***(i-**1**)+**1**;

value=**1**;

output;

parm=**5**;

row=**3***(i-**1**)+**1**;

col=**3***(i-**1**)+**3**;

value=**1**;

output;

parm=**5**;

row=**3***(i-**1**)+**3**;

col=**3***(i-**1**)+**1**;

value=**1**;

output;

parm=**6**;

row=**3***(i-**1**)+**2**;

col=**3***(i-**1**)+**3**;

value=**1**;

output;

parm=**6**;

row=**3***(i-**1**)+**3**;

col=**3***(i-**1**)+**2**;

value=**1**;

output;

end;

drop i;

**run**;

/* Functional Group Model with RE in all years (REs correlated) */

**proc** **mixed** data=RANDOMDI.data_1000_300 method=reml noprofile scoring = **10** maxiter = **100**;

where count = **1**;

by count;

class year plot;

model Y = P1*year P2*year P3*year P4*year P5*year P6*year P7*year P8*year P9*year P10*year PPwfg1*year PPwfg2*year PPbfg12*year / noint solution;

repeated year / subject=plot type=cs;

random Y1PP1 Y2PP1 Y3PP1 Y1PP2 Y2PP2 Y3PP2 Y1PP3 Y2PP3 Y3PP3 Y1PP4 Y2PP4 Y3PP4 Y1PP5 Y2PP5 Y3PP5 Y1PP6 Y2PP6 Y3PP6 Y1PP7 Y2PP7 Y3PP7 Y1PP8 Y2PP8 Y3PP8 Y1PP9 Y2PP9 Y3PP9 Y1PP10 Y2PP10 Y3PP10

Y1PP11 Y2PP11 Y3PP11 Y1PP12 Y2PP12 Y3PP12 Y1PP13 Y2PP13 Y3PP13 Y1PP14 Y2PP14 Y3PP14 Y1PP15 Y2PP15 Y3PP15 Y1PP16 Y2PP16 Y3PP16 Y1PP17 Y2PP17 Y3PP17 Y1PP18 Y2PP18 Y3PP18 Y1PP19 Y2PP19 Y3PP19 Y1PP20 Y2PP20 Y3PP20

Y1PP21 Y2PP21 Y3PP21 Y1PP22 Y2PP22 Y3PP22 Y1PP23 Y2PP23 Y3PP23 Y1PP24 Y2PP24 Y3PP24 Y1PP25 Y2PP25 Y3PP25 Y1PP26 Y2PP26 Y3PP26 Y1PP27 Y2PP27 Y3PP27 Y1PP28 Y2PP28 Y3PP28 Y1PP29 Y2PP29 Y3PP29 Y1PP30 Y2PP30 Y3PP30

Y1PP31 Y2PP31 Y3PP31 Y1PP32 Y2PP32 Y3PP32 Y1PP33 Y2PP33 Y3PP33 Y1PP34 Y2PP34 Y3PP34 Y1PP35 Y2PP35 Y3PP35 Y1PP36 Y2PP36 Y3PP36 Y1PP37 Y2PP37 Y3PP37 Y1PP38 Y2PP38 Y3PP38 Y1PP39 Y2PP39 Y3PP39 Y1PP40 Y2PP40 Y3PP40

Y1PP41 Y2PP41 Y3PP41 Y1PP42 Y2PP42 Y3PP42 Y1PP43 Y2PP43 Y3PP43 Y1PP44 Y2PP44 Y3PP44 Y1PP45 Y2PP45 Y3PP45 / type=lin(**6**) ldata=RANDOMDI.re3 solution;

**run**;

**S.11: Total plot intraspecific interactions split by spatial pattern (aggregated or dispersed) for each level of richness (2, 4, 8).**


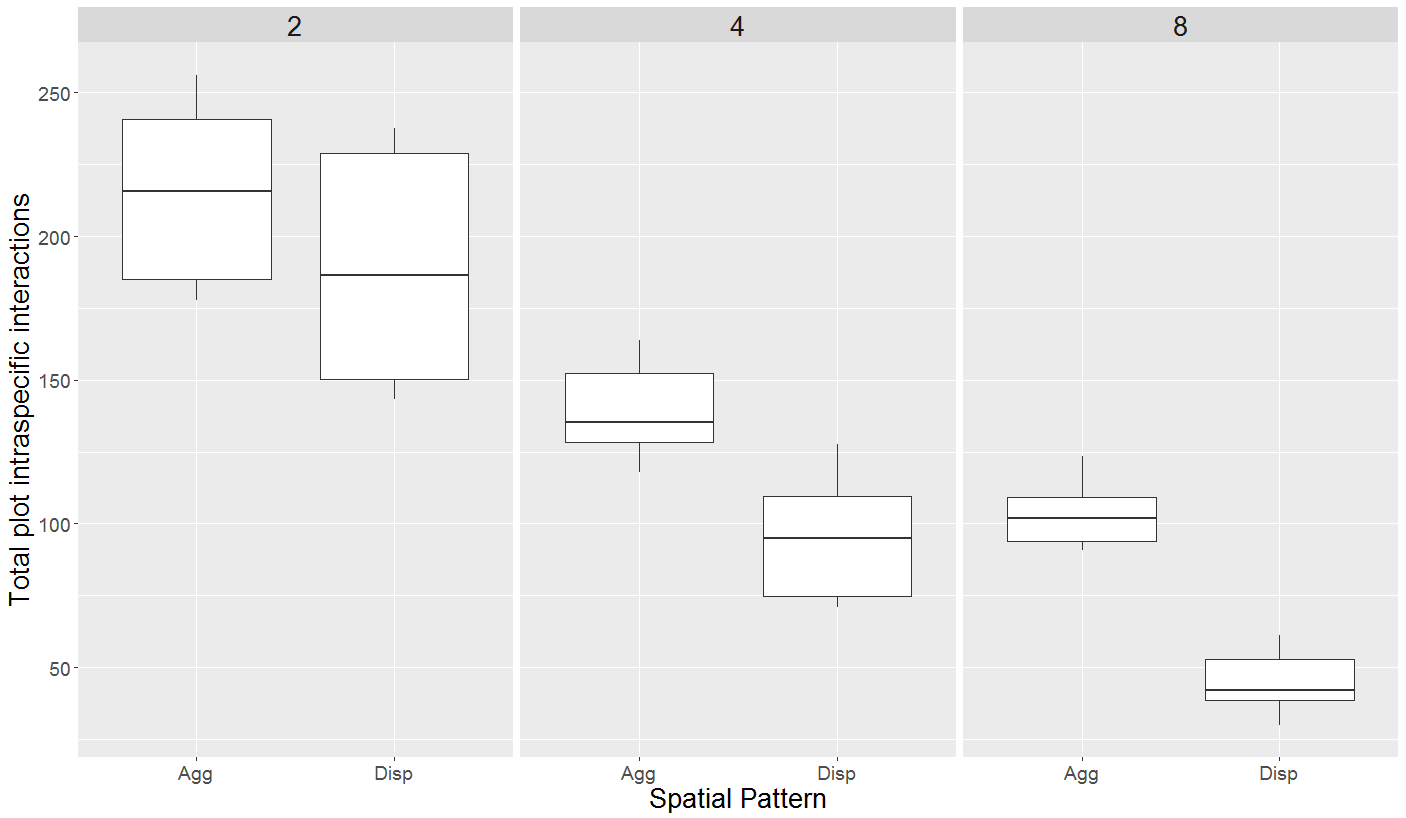


Figure 6: Total plot intraspecific interactions split by spatial pattern (aggregated or dispersed) for each level of richness (2, 4, 8). The intraspecific interaction for each species in the plot is the sum of the inverse squared distance between each of the cells containing the species, where adjacent species are a distance of 1 apart. The total plot intraspecific interaction is the sum of the intraspecific interactions for all species in the plot
